# Examination of the Effects of Heterogeneous Organization of RyR Clusters, Myofibrils and Mitochondria on Ca^2+^ Release Patterns in Cardiomyocytes

**DOI:** 10.1371/journal.pcbi.1004417

**Published:** 2015-09-03

**Authors:** Vijay Rajagopal, Gregory Bass, Cameron G. Walker, David J. Crossman, Amorita Petzer, Anthony Hickey, Ivo Siekmann, Masahiko Hoshijima, Mark H. Ellisman, Edmund J. Crampin, Christian Soeller

**Affiliations:** 1 Department of Mechanical Engineering, University of Melbourne, Melbourne, Australia; 2 Auckland Bioengineering Institute, University of Auckland, Auckland, New Zealand; 3 Systems Biology Laboratory, Melbourne School of Engineering, University of Melbourne, Melbourne, Australia; 4 Department of Engineering Science, University of Auckland, Auckland, New Zealand; 5 Department of Physiology, University of Auckland, Auckland, New Zealand; 6 School of Biological Sciences, University of Auckland, Auckland. New Zealand; 7 Department of Medicine, University of California San Diego, San Diego, United States of America; 8 National Center for Microscopy and Imaging Research, University of California San Diego, San Diego, United States of America; 9 School of Mathematics and Statistics, Faculty of Science, University of Melbourne, Melbourne, Australia; 10 School of Medicine, Faculty of Medicine, Dentistry and Health Sciences, University of Melbourne, Melbourne, Australia; 11 ARC Centre of Excellence in Convergent Bio-Nano Science and Technology, University of Melbourne, Melbourne, Australia; 12 Biomedical Physics, University of Exeter, Exeter, United Kingdom; University of Michigan, UNITED STATES

## Abstract

Spatio-temporal dynamics of intracellular calcium, [Ca^2+^]_i_, regulate the contractile function of cardiac muscle cells. Measuring [Ca^2+^]_i_ flux is central to the study of mechanisms that underlie both normal cardiac function and calcium-dependent etiologies in heart disease. However, current imaging techniques are limited in the spatial resolution to which changes in [Ca^2+^]_i_ can be detected. Using spatial point process statistics techniques we developed a novel method to simulate the spatial distribution of RyR clusters, which act as the major mediators of contractile Ca^2+^ release, upon a physiologically-realistic cellular landscape composed of tightly-packed mitochondria and myofibrils. We applied this method to computationally combine confocal-scale (~ 200 nm) data of RyR clusters with 3D electron microscopy data (~ 30 nm) of myofibrils and mitochondria, both collected from adult rat left ventricular myocytes. Using this hybrid-scale spatial model, we simulated reaction-diffusion of [Ca^2+^]_i_ during the rising phase of the transient (first 30 ms after initiation). At 30 ms, the average peak of the simulated [Ca^2+^]_i_ transient and of the simulated fluorescence intensity signal, F/F_0_, reached values similar to that found in the literature ([Ca^2+^]_i_ ≈1 μM; F/F_0_≈5.5). However, our model predicted the variation in [Ca^2+^]_i_ to be between 0.3 and 12.7 μM (~3 to 100 fold from resting value of 0.1 μM) and the corresponding F/F_0_ signal ranging from 3 to 9.5. We demonstrate in this study that: (i) heterogeneities in the [Ca^2+^]_i_ transient are due not only to heterogeneous distribution and clustering of mitochondria; (ii) but also to heterogeneous local densities of RyR clusters. Further, we show that: (iii) these structure-induced heterogeneities in [Ca^2+^]_i_ can appear in line scan data. Finally, using our unique method for generating RyR cluster distributions, we demonstrate the robustness in the [Ca^2+^]_i_ transient to differences in RyR cluster distributions measured between rat and human cardiomyocytes.

## Author Summary

Calcium (Ca^2+^) acts as a signal for many functions in the heart cell, from its primary role in triggering contractions during the heartbeat to acting as a signal for cell growth. Cellular function is tightly coupled to its ultra-structural organization. Spatially-realistic and biophysics-based computational models can provide quantitative insights into structure-function relationships in Ca^2+^ signaling. We developed a novel computational model of a rat ventricular myocyte that integrates structural information from confocal and electron microscopy datasets that were independently acquired and includes: myofibrils (protein complexes that contract during the heartbeat), mitochondria (organelles that provide energy for contraction), and ryanodine receptors (RyR, ion channels that release the Ca^2+^ required to trigger myofibril contraction from intracellular stores). Using this model, we examined [Ca^2+^]_i_ dynamics throughout the cell cross-section at a much higher resolution than previously possible. We estimated the size of structural maladaptation that would cause disease-related alterations in [Ca^2+^]_i_ dynamics. Using our methods for data integration, we also tested whether reducing the density of RyRs in human cardiomyocytes (~40% relative to rat) would have a significant effect on [Ca^2+^]_i_. We found that Ca^2+^ release patterns between the two species are similar, suggesting Ca^2+^ dynamics are robust to variations in cell ultrastructure.

## Introduction

The cardiac myocyte possesses a highly organized assembly of membrane networks, contractile proteins, ion channels and buffering systems. The heartbeat is the result of tightly regulated electrical, chemical, and mechanical processes that repeatedly occur at the subcellular scale across the millions of cells that make up the heart—a phenomenon called excitation-contraction coupling (ECC) [1–3]. ECC begins with the electrical activation and depolarization of the cell membrane and its transverse-tubular extensions (t-tubules) that invaginate the inner depths of the cell, thus causing a small flux of Ca^2+^ (through voltage-dependent L-type Ca^2+^ channels) into the dyadic cleft—the space between the t-tubules and the extensive internal Ca^2+^ storage network called the sarcoplasmic reticulum (SR). This Ca^2+^ triggers the opening of a set of ion channels located on the SR called the ryanodine receptors (RyRs) causing a large flux of Ca^2+^ to enter the dyadic cleft from the SR. Finally, the resulting increase in intracellular Ca^2+^ concentration ([Ca^2+^]_i_) activates the surrounding contractile machinery, thus effecting a contractile response.

We aim to investigate the effect that experimentally measured spatial distributions of myofibrils, mitochondria, and RyR clusters have on Ca^2+^ release and diffusion in the cardiac cytosol. Structural imaging using confocal, super-resolution, and electron microscopy [4–11] are providing insights into the structural organization of the cardiac cell at different spatial scales. These datasets are increasingly being used to generate accurate, spatially-extended computational models of the cell in order to investigate intracellular Ca^2+^ dynamics that current [Ca^2+^]_i_ imaging technologies cannot resolve [12–15]. However, because these models are generated from a single structural dataset, the spatial resolution of the model and the different organelle components included are restricted by the spatial resolution and organelle components available in the structural data. In addition, the robustness of these computational models to variations in structural organization is hard to assess, since the model geometry is strongly dependent on the datasets at hand.

This study presents a new method for generating a spatially accurate 3D computational model of cardiac cell myofibrils, mitochondria, and clusters of ryanodine receptors. The method uses spatial statistics techniques [16,17] to characterize and model the spatial distribution of RyR clusters relative to the contractile machinery. The statistical model then enables the fusion of data on the spatial distribution of RyR clusters from high-contrast confocal-resolution images (~ 200 nm) with data on the organization of myofibrils and mitochondria available from 3D electron microscopy images (to a resolution of ~30 nm). Using this method, we generated a 3D half-sarcomere spatial model of the organization of myofibrils, mitochondria, and RyR clusters in an adult Wistar rat left ventricular cardiomyocyte.

Inhomogeneities in the spatio-temporal dynamics in the first 30 ms—the rising phase—of the [Ca^2+^]_i_ transient have been examined and used as a measure of t-tubule degradation (and other disease-related structural remodeling events) in many previous studies on structure-function relationships in cellular cardiology and heart failure [18–21]. Spatio-temporal variations in the rising [Ca^2+^]_i_ transient could result in reduced contractile power. Furthermore, cellular processes such as the calcineurin-NFAT pathway to cardiac hypertrophy, apoptosis, stress-response, and energy metabolism are [Ca^2+^]_i_ dependent [21]. Therefore, using this model, we simulated the rising phase of the [Ca^2+^]_i_ transient—using biophysical equations to account for Ca^2+^ buffering and Ca^2+^ indicator-dye kinetics—and assessed two aspects of the relationship between cell structure and [Ca^2+^]_i_ dynamics: (i) the effect of the realistic spatial organization of myofibrils, mitochondria, and RyR clusters on the spatial heterogeneity of [Ca^2+^]_i_; (ii) and the sensitivity of these effects to variations in RyR cluster organization that have been measured in previous experimental studies [8,9,22].

We further demonstrate the power of this structurally realistic model in systematically assessing the degree of heterogeneity that can be introduced in the cell-wide Ca^2+^ transient by the arrangement of the cell’s contractile machinery and Ca^2+^ release sites. Specifically, we explored the degree to which structure-induced heterogeneities in [Ca^2+^]_i_, that we predicted computationally, would be visible in simulated line-scan images.

Our model showed that: (i) spatial heterogeneity in [Ca^2+^]_i_ can partly be caused by the non-uniform distribution and clustering of mitochondria in relation to the myofibrils and the sarcolemma; (ii) spatial heterogeneity in [Ca^2+^]_i_ can also be caused by localized aggregation of RyR clusters observed in the structural data; (iii) variations in RyR cluster organization could give rise to observable differences in line scan data; and (iv) differences in the spread and density of RyR clusters between different mammalian species (as reported in the literature) have only a marginal effect on [Ca^2+^]_i_ at the peak of the transient, thus highlighting the robustness of the ECC system to structural variations in RyR cluster distributions. Furthermore, our mathematical model showed that in order to observe a reduction in regional fluorescence in line scan images (as reported in studies on disease-related alterations to t-tubules and RyR cluster activity), RyR clusters within a 1.29 μm diameter neighborhood (corresponding to 4 to 6 RyR clusters) must remain un-activated in an action-potential evoked transient.

The following sections present the results of the [Ca^2+^]_i_ simulations on the hybrid-scale spatial model that were analyzed to derive the above stated findings. The basic study design and the biophysical model are outlined in the materials and methods. The method for generating the hybrid model and the biophysical equations for the simulations are detailed in S1 and S2 Texts, respectively. We make special note that we have made the hybrid-scale geometric model and [Ca^2+^]_i_ simulation codes available at our publically accessible repository: https://github.com/vraj004/cardiac_ecc. We have also made the RyR cluster simulation algorithm and the experimental data that were used to develop the algorithm and simulations available at: https://github.com/vraj004/RyR-simulator. We encourage cardiac computational scientists to make use of our resources in their research of cardiac biophysics at the subcellular scale. We conclude the study with a discussion of the limitations of the current analysis and future directions.

## Results

### A 3D model of RyR clusters, mitochondria and myofibrils through computational fusion of 3D electron tomography and confocal microscopy data

**Fig 1:**
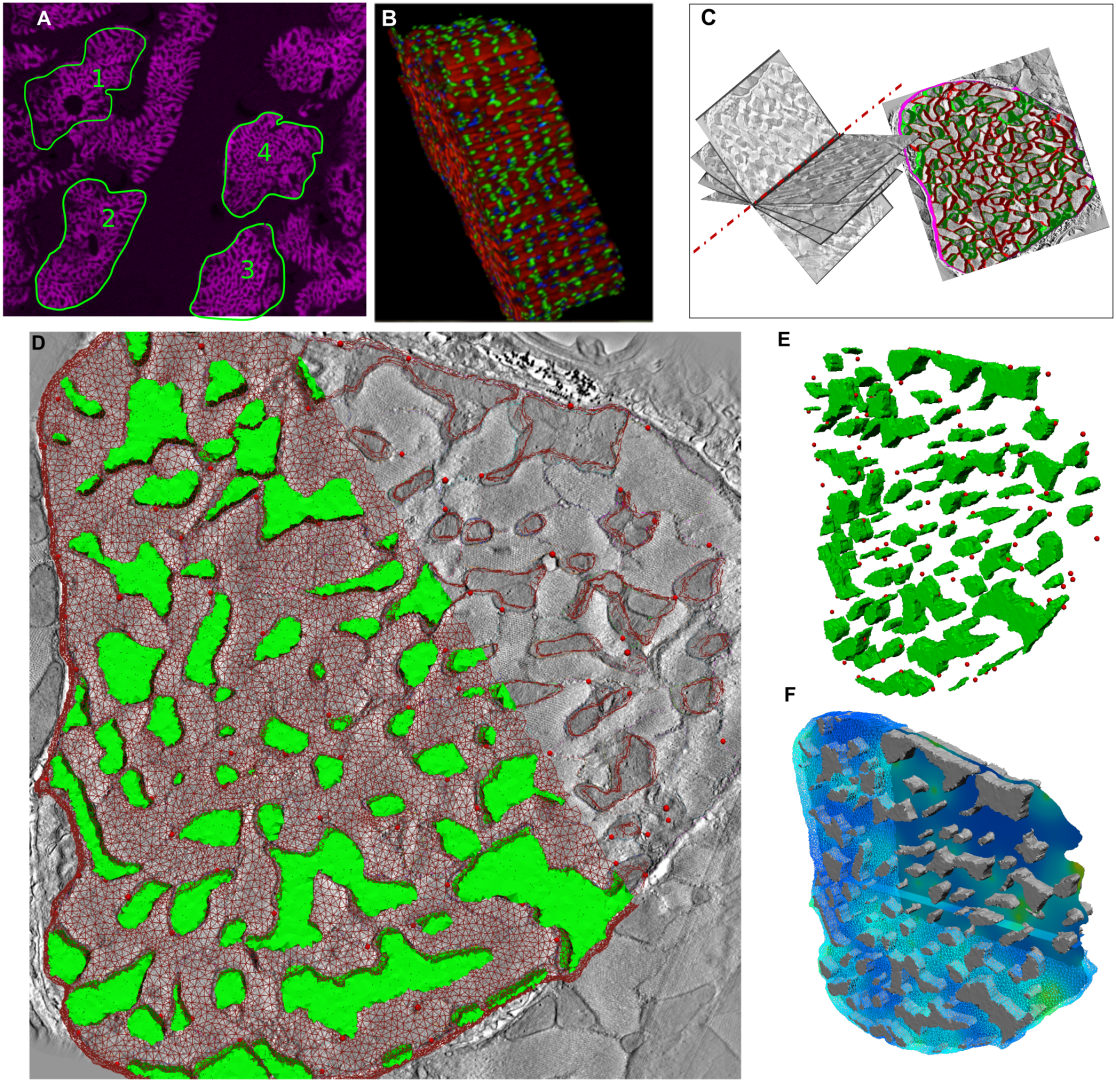
A hybrid-scale spatial model of myofbirils, mitochondria and RyR clusters. (A): Confocal image of a tissue section from the left ventricule of an adult male Wistar rat; numbered cells were processed for RyR cluster distribution analysis and development of a novel computational fusion algorithm (see S1 Text). (B): 3D rendering of cell number 1 in (A) showing green immuno-labeling of RyR clusters and red, phalloidin staining of myofibrillar actin. (C): Electron micrographs of a 240 nm tissue section from another left ventricular sample of a similar male Wistar rat were acquired at different tilts (left) to construct a 3D electron tomogram; the stack was manually segmented (right) for myofibrils and mitochondria. More details on the data acquisition are in the Materials and Methods. (D): One image slice from the electron tomogram with an overlay of the FE computational mesh (mitochondrial regions in green) and the simulated RyR clusters (red spheres) from the computational fusion algorithm; the mesh is partly removed for visualization. (E): A 3D view of the RyR clusters and the mitochondrial regions. (F): 3D view of the predicted Fluo-4-bound Ca^2+^ at the end of 30 ms; an isosurface of the solution field is partially in view at the mid-plane of the half-sarcomere model.

Confocal microscopy has provided several important insights on the organization of RyR clusters and their Ca^2+^ release kinetics that have helped develop several quantitative models of Ca^2+^ biophysics [29–31]. One aim of this study was to explore the effect that the structural organization of RyR clusters around the contractile machinery and mitochondria had on spatio-temporal dynamics of [Ca^2+^]_i_; current spatially extended models of cardiac cell structure use simplified representations of the organization of myofibrils and mitochondria [32–36]. Furthermore, although myofibrils and mitochondria can be visualized under confocal microscopes (with appropriate antibody labels), the light diffraction limit prevents accurate segmentation of myofibril bundles and mitochondria [7]. Therefore, we aimed to explore [Ca^2+^]_i_ dynamics in a geometric model that was neither limited by the spatial resolution of confocal microscopy (with regards to the contractile machinery and mitochondria), nor by the lack of contrast and small field of view that is inherent in electron microscopy (and therefore makes cell-wide RyR cluster visualization infeasible). We developed a novel algorithm that generates realistic confocal-scale RyR cluster distributions around nanometer-resolution 3D templates of myofibril and mitochondrial organization acquired using 3D electron tomography. This approach enabled us to create a hybrid-scale spatial model of the distribution of myofibrils, mitochondria, and RyR clusters (see Fig 1) that can be used with biophysical models of Ca^2+^ release and buffering to examine the dynamics of [Ca^2+^]_i_ at the nanometer scale.

Three key observations could be made from the analysis of the spatial distribution of RyR clusters and the development of the algorithm that enable simulations of realistic distributions of RyR clusters on any given organization of myofibrils and mitochondria:

1. The number of clusters in a cell and the nearest-neighborhood distance distribution are consistent (0.59±0.16 μm) between each of the cells we studied (see S1 Text, S1 and S2 Tables and S9 Fig). These metrics are also consistent with reported metrics from previous studies [8,9,23]
2. We tested the hypothesis that an algorithm will generate RyR cluster distributions that are similar in characteristics to experimentally measured spatial distributions observed in a given cell, provided that the simulated nearest-neighborhood distribution and the number of RyR clusters matched the experimentally measured values in the cell (S10 and S11 Figs).
3. Given, from (1), that the nearest-neighborhood distance and number of clusters are consistent across cells, we also showed that these two parameters were sufficient to recapitulate distributions of RyR clusters in all of the cell geometries we had imaged using the confocal microscope; the specific distribution of myofibrils within each cell does not affect the accuracy of the RyR cluster simulation.

For the particular tomogram that we used as a template of high-resolution myofibril and mitochondrial organization (Fig 1C and 1D), the cross-sectional area of the cell is approximately 94.8 μm^2^. With an average density of 1.3 couplons/μm^2^ of cell-cross-section (based on measured densities across z-discs in confocal datasets, S2 Table), we simulated 123 RyR clusters on the tomogram-derived template cross-section. The tomogram cross-section was extruded to create a half-sarcomere computational model (see Materials and Methods and S2 Fig) of RyR clusters, mitochondria, and myofibrils that was spatially consistent with structural imaging data (Fig 1D and 1E). Further details of the RyR simulation steps and the generation of the computational model are provided in the Materials and Methods section and in S1 Text.

Unlike studies that simulate the distribution of RyR clusters as regularly spaced release sites of Ca^2+^ [32,34,35], close examinations of Fig 1D shows clusters are not regularly spaced. These structural heterogeneities in the distribution of Ca^2+^ release sites can have an impact on the spatial dynamics of [Ca^2+^]_i_. In addition, unlike existing models that incorporate idealized, regularly spaced representations of the contractile machinery and mitochondria [32–33], our model captures the experimentally observed heterogeneity as seen in Fig 1C. The following biophysical simulations of Ca^2+^ release and diffusion examine the role that these structural heterogeneities may play in the spatiotemporal dynamics of [Ca^2+^]_i_.

### Spatial heterogeneity in [Ca^2+^]_i_ during the rising phase of the Ca^2+^ transient

Details of the computational model and the biophysical equations are described in Materials and Methods and S2 Text. Briefly, the finite element model in Fig 1 is composed of 222,312 nodes making up 1,307,928 trilinear simplex tetrahedral elements. Computational nodes within a 100 nm radius of the simulated positions of RyR clusters were assigned as Ca^2+^ source nodes. 2 pA of Ca^2+^ was injected into the cell at each of the RyR cluster zones using a realistic Ca^2+^ release profile similar to previous studies 29,31,32] (see S3 Fig). Along with the spatio-temporal dynamics of [Ca^2+^]_i_, the diffusion of Fluo-4 (F4) and Ca^2+^ bound Fluo-4 (F4Ca) were also simulated as coupled partial differential equations to simulate Ca^2+^-dye binding kinetics and diffusion. Troponin C was distributed homogeneously through the myofibrillar space and acted as a stationary buffer of the diffusing Ca^2+^. We restricted the simulations to incorporate minimal RyR cluster gating kinetics (for example, calcium-induced calcium release was not included) and minimal Ca^2+^ buffering components to explore the sensitivity of [Ca^2+^]_i_ dynamics to structural organization alone. The simulations were conducted for the first 30 milliseconds (ms) of the ECC cycle, representing the rising phase of the Ca^2+^ transient. The remaining, re-uptake phase of the ECC cycle was not considered in this study because our aim was to explore the role of the spatially realistic organization of the included components of the cell; modeling the re-uptake phase would require, among other things, a realistic distribution of sarco-endoplasmic reticulum calcium pumps (SERCA). The initiation of release from each RyR cluster was stochastically varied to simulate the experimentally observed stochasticity in the coupling latency between RyR clusters and L-type Ca^2+^ channels [31,37]. The biochemical rates and initial concentrations of [Ca^2+^]_i_ and other buffer components are outlined in Table 1 and were based on published values [29,38].

**Table 1:** Biochemical rate constants, diffusion constants and initial concentrations used for the Ca^2+^ simulations.

Resting [Ca^2+^]_i_	0.1uM
V_cyto_	4.19x10^-3^ μm^3^
Dcyto [Ca^2+^]	0.22 μm^2^/ms
Dmito [Ca^2+^]_i_	0.0 μm^2^/ms
Dcyto [F4Ca]_i_	0.042μm^2^/ms
Dmito [F4Ca]_i_	0.0 μm^2^/ms
iCa, RyR cluster Ca^2+^ release current	2 e-15 C/ms (2 pA)
F, Faraday’s constant	96500 C/mol
Total [F4]	25 μM
[F4]_i_ initial	22.92 μM
[F4Ca]_i_ initial	2.08 μM
K_D_ F4Ca	1.1 μM
F4 k_i_^off^	0.11 /ms
F4 k_i_^on^	0.1 /μMms
Total [Troponin C]	70 μM
K_D_ Ca-Troponin C	0.6 μM
[Ca-Troponin C]_0_	10 μM
[TroponinC]_0_	60 μM
Troponin C k_i_^off^	0.0196 /ms
Troponin C k_i_^on^	0.0327 /μMms

**Fig 2:**
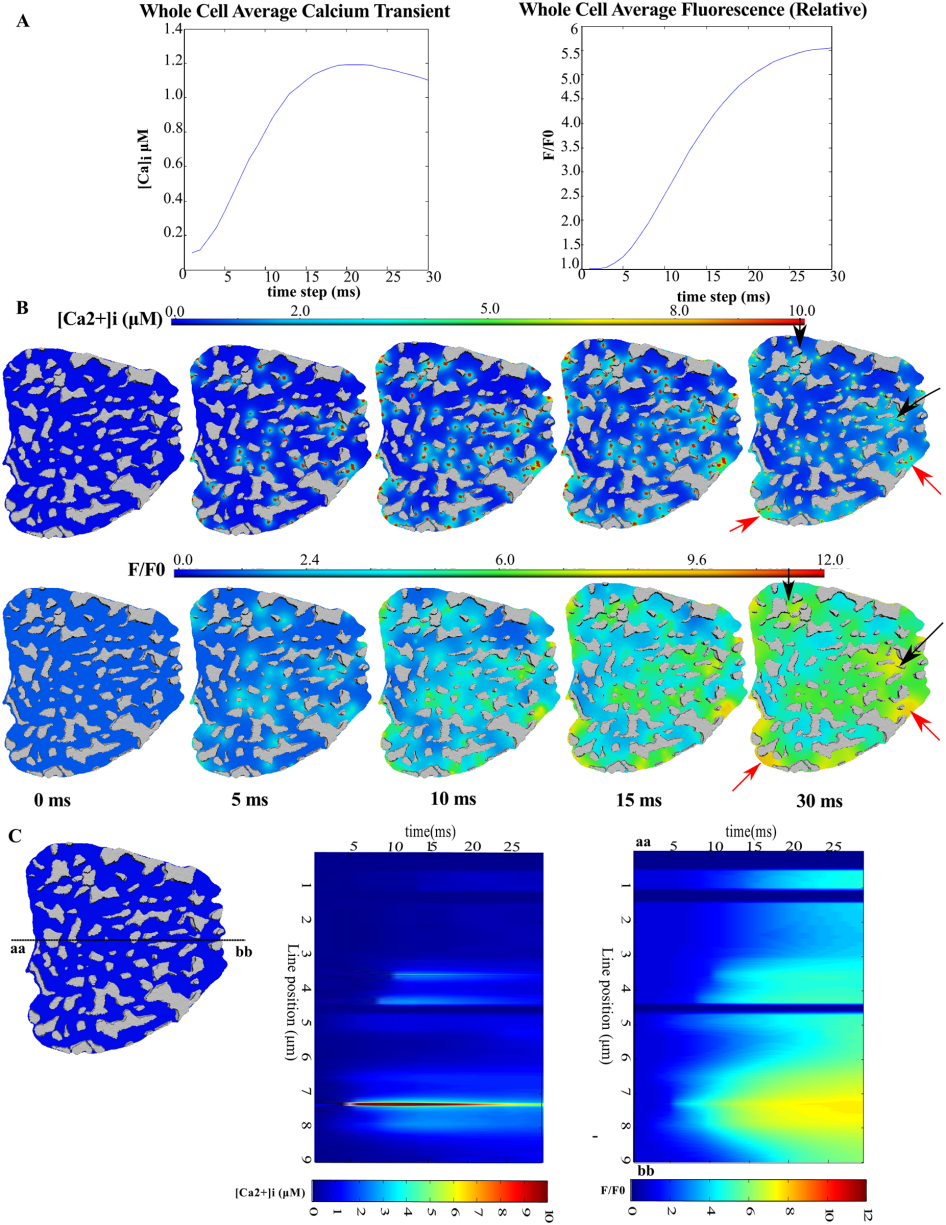
Spatial heterogeneity in [Ca^2+^]_i_ during the rising phase of the Ca^2+^ transient. (A): The average cytosolic freely diffusing [Ca^2+^]_i_ (left) and Fluo-4 bound Ca^2+^, [F4Ca]_i_ (right). (B) and (C) show time-lapse snapshots of freely diffusing Ca^2+^ and F4Ca at the z-disc, transverse plane; black arrows mark regions of high [Ca^2+^]_i_ due to mitochondrial clustering; red arrows mark regions of high [Ca^2+^]_i_ due to several ryr clusters in close proximity. (C): simulated line scans of [Ca^2+^]_i_ (middle) and [F4Ca]_i_ (right). The line position is shown on the cell cross-section on the left.

Fig 2 and S1 and S2 Movies show the spatio-temporal dynamics of freely diffusing [Ca^2+^]_i_ and [F4Ca]_i_ at the z-disc of the half-sarcomere model. Fig 2A shows that the average cytosolic [Ca^2+^]_i_ transient rises to approximately 1 μM and the relative F4Ca transient (F/F_0_) rises approximately 5-fold. These values are within the range of many experimental measurements of Ca^2+^ transients in healthy rat ventricular myocytes found in the literature [22,24–28].

Fig 2B shows time-lapse snapshots of the freely diffusing Ca^2+^ and F4Ca. At the end of 30 ms, [Ca^2+^]_i_ has range of values between 0.3 and 12.7 μM; the F4Ca F/F_0_ signal ranges from 3 to 9.5. Careful examination of the 30 ms snapshots of the [Ca^2+^]_i_ and [F4Ca]_i_ signals shows several features that give rise to “hot-spots”. Some hot-spots occur due to the close proximity of several RyR clusters (red arrows), while others occur due to Ca^2+^ release into regions bounded by clusters of mitochondria (black arrows).

Fig 2C shows line-scans of [Ca^2+^]_i_ (middle) and [F4Ca]_i_ (right) taken along the line-position on the left figure. The peak [Ca^2+^]_i_ of ~10 μM is close to a release site and is of a magnitude similar to that reported in the literature previously [29–31]. Dark blue strips represent the inside of mitochondria; we assumed that intra-mitochondrial Ca^2+^ is only marginally affected by the Ca^2+^ transient and that the mitochondrial uniporter plays a negligible role as a cytosolic Ca^2+^ buffer [27,39,40].

We examined whether the spatial pattern of [Ca^2+^]_i_ and [F4Ca]_i_ at 30 ms was also a function of the stochasticity in RyR cluster triggering. With the given RyR cluster distribution and mesh in Fig 1, four different sets of RyR cluster opening times were sampled from an exponential distribution with a characteristic decay constant of 6.7 ms (see S2 Text). S4 Fig shows that at the end of the 30 ms, the spatial pattern of [F4Ca]_i_ (and [Ca^2+^]_i_ not shown) is not dependent on the release times of the RyR clusters. Instead, the spatial pattern is characteristic of the organization of the cell components in the mesh.

**Fig 3:**
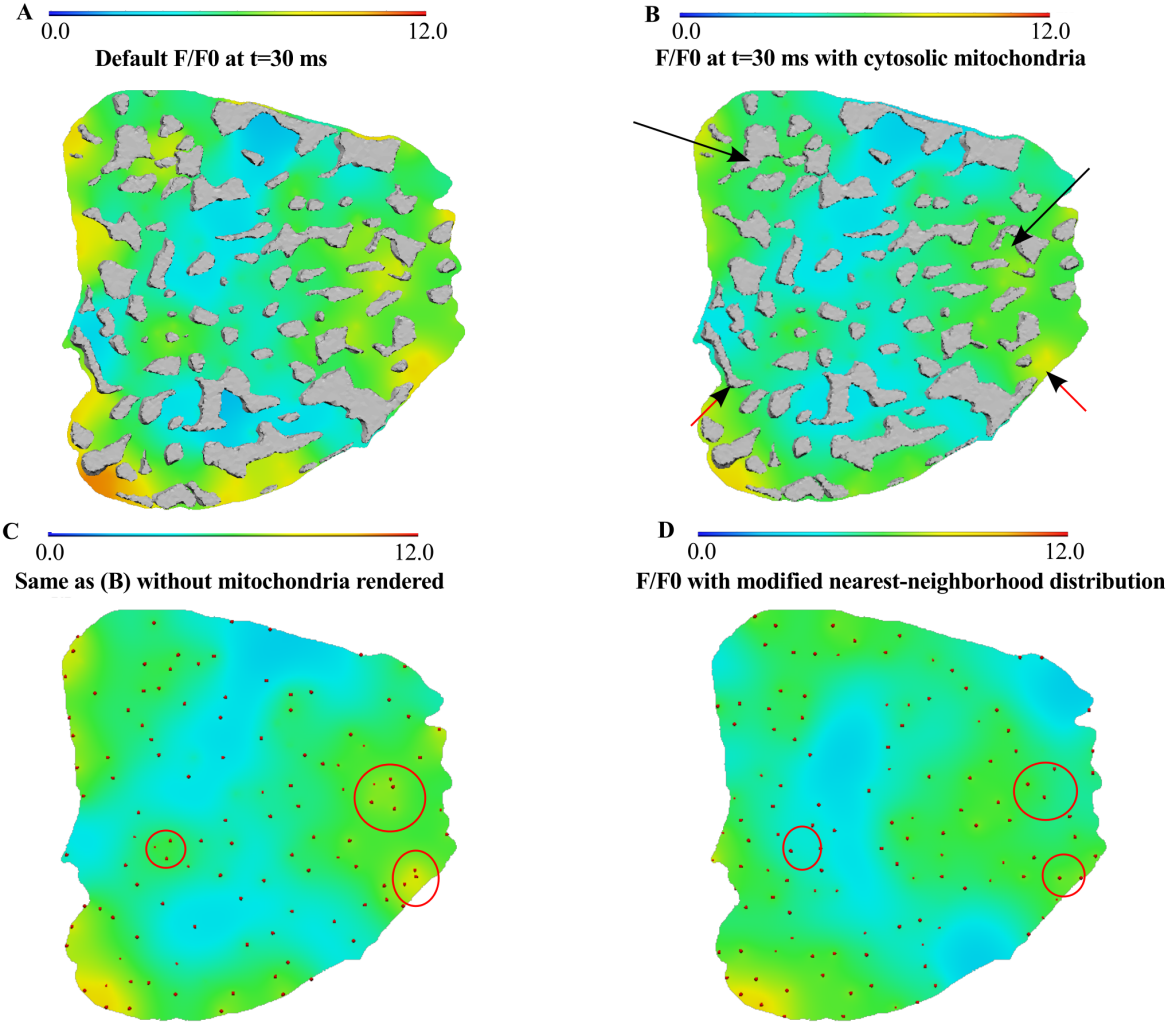
The impact of mitochondria and RyR cluster distribution on the [Ca^2+^]_i_ transient. (A): The F/F_0_ signal from Fig 2C. (B) F/F_0_ signal with mitochondrial regions being assigned cytosolic properties. (C) same as (B) without mitochondria rendered. The red dots illustrate the RyR cluster distribution that matches experimental data. (D) Changed F/F_0_ signal when the RyR cluster distribution is forced to have a fixed nearest-neighbor distance between all clusters.

We finally illustrate the impact that heterogeneous distributions of mitochondria and RyR clusters have on the heterogeneity of the [Ca^2+^]_i_ transient. Fig 3 shows the F/F_0_ signal at 30 ms (A) with the default simulation settings; (B-C) with mitochondrial regions replaced with cytosolic properties; and (D) with RyR clusters being forced to have a nearest-neighbor at the mean nearest-neighborhood distance that was measured experimentally, but with zero variance as opposed to being distributed with nearest-neighborhood distances of the experimentally measured mean and variance. Note that (C) shows (B) without the mitochondria rendered and clearly shows the increase in homogeneity in the F/F_0_ signal. The red points depict the distribution of RyR clusters that are similar in characteristics to experimental data. (D) shows the effect of imposing a regular distribution of RyR clusters—by way of positioning nearest-neighbors of every cluster at a fixed distance. The encircled regions show that the F/ F_0_ signal is less intense where RyR clusters are less closely positioned. These results thus confirm that structural heterogeneities in the cell can introduce spatial heterogeneities in [Ca^2+^]_i_.

Similar to previous studies [15], we examined the effect of buffering by Fluo-4 by simulating [Ca^2+^]_i_ release and diffusion in the absence of Fluo-4. S5 Fig shows that without Fluo-4, bulk [Ca^2+^]_i_ increases and the heterogeneous distribution of [Ca^2+^]_i_ is not greatly affected. However, clusters of RyRs that are in close proximity of each other amplify the hot-spots more because of the lack of buffering by Fluo-4.

### The specific distribution of RyR clusters also affects the locations of high [Ca^2+^]_i_ in the rising transient

**Fig 4:**
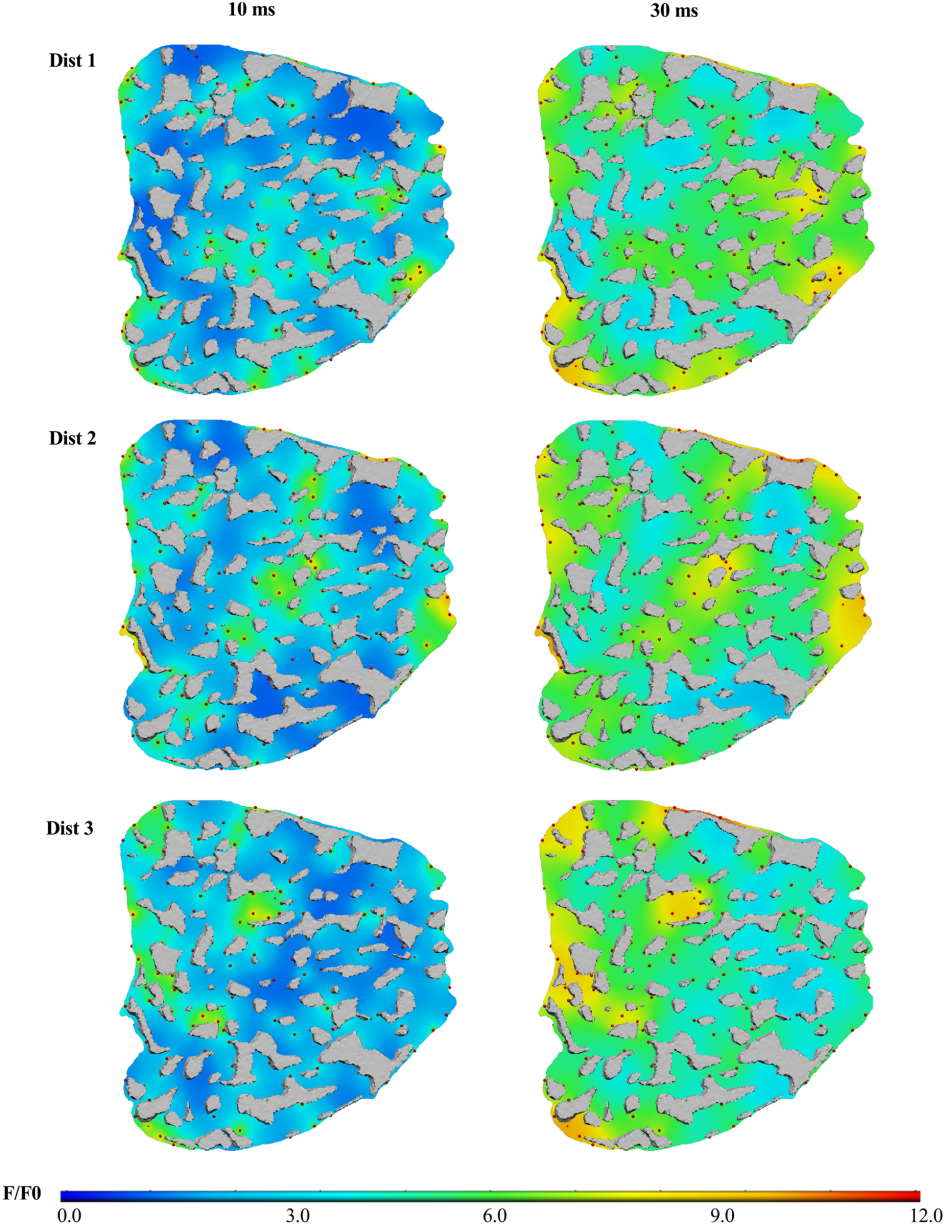
The specific distribution of RyR clusters affects the locations of high [Ca^2+^]_i_. Snapshots of [F4Ca]_i_ at t = 10 ms (left) and t = 30 ms (right) are shown for three different RyR cluster distributions; spheres in red mark centroids of simulated RyR clusters; grey regions mark mitochondria.

The computational algorithm for generating RyR clusters can be used to simulate many possible distributions of RyR clusters that fall into the family of statistically equivalent distributions to the experimentally observed data. We tested whether different RyR cluster distributions from the family of possible distributions would affect the bulk cytosolic transients or the spatial heterogeneity of [Ca^2+^]_i_. Fig 4 shows that different RyR cluster distributions do not alter the net [Ca^2+^]_i_ transient, but affect where “hot-spots” in [Ca^2+^]_i_ arise. As such, the RyR cluster algorithm provides valid RyR cluster distributions. Furthermore, the result shows that the distribution of RyR clusters (in addition to organization of mitochondria) also affect the spatial pattern of [Ca^2+^]_i_. These results have not been captured in previous modeling studies [14,32,35] because this is the first model that accurately captures realistic spatial distributions of RyR clusters as well as the mitochondria and myofibrils.

### Varying RyR cluster distribution can introduce observable differences in heterogeneities in line scan data

The line scan of the F/F_0_ signal in Fig 2C shows marked heterogeneity compared to any line scan that is available in literature [12–15]. Cannell et.al [41] examined non-uniformities in line scan plots that were oriented in the longitudinal direction of the cell and inferred that some of the heterogeneity in the rising transient could be explained by the presence of non-ECC components such as mitochondria. We have shown in greater detail, albeit in the transverse direction, that heterogeneities not only arise from the mitochondria, but can also arise from heterogeneities in the positioning of Ca^2+^ release sites and confined spaces created by groups of mitochondria or the sarcolemma. Here we aimed to quantify the degree to which the heterogeneities we have predicted would contribute to the heterogeneity in the rising transient observed in line scan plots.

**Fig 5:**
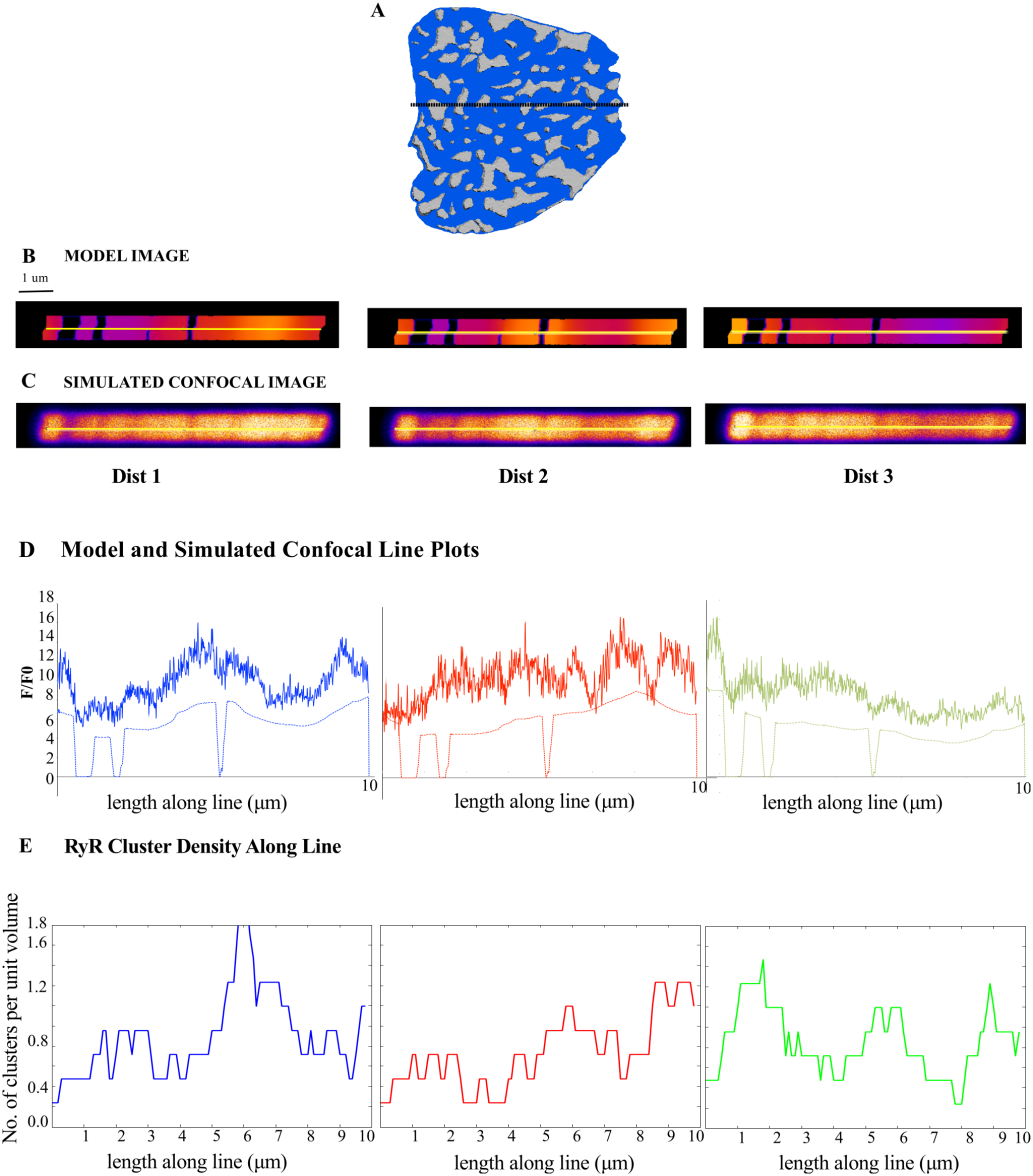
Structure-induced spatial heterogeneity at line scan resolution. (A): Illustration marking depth through the cross-section where the [Ca^2+^]_i_ transient was visualized for line scan image simulations. (B): Model view of [F4Ca]_i_ transverse to the cross-section of the cell visualized in (A) at the line scan depth at t = 30 ms for three different RyR cluster distributions (labelled Dist 1, Dist 2 and Dist 3). (C): the model images were convolved with a confocal microscope point-spread-function and multiplied with poisson noise to generate simulated confocal images for the three RyR cluster distributions. (D): Line plots from the model (dashed-lines) and confocal simulated views (bold lines) show variation in [F4Ca]_i_ along the yellow lines (shown in B and C). (E): A plot of the density of RyR clusters, for each RyR cluster distribution, in a 1 micron spherical neighborhood along the length of the yellow lines in (B and C). A comparison of (E) against (D) shows that RyR cluster organization can introduce heterogeneity in line scans.

Fig 5B shows the F/F_0_ signal on the plane transverse to the cell cross-section at the line scan depth shown in Fig 5A for t = 30 ms; line scans images were generated for the three RyR cluster distributions in Fig 4. The transverse-plane [F4Ca]_i_ (Fig 5B, Model Image Panel), visualized with a modified colour look up table, shows gaps corresponding to mitochondria, as well as spatial heterogeneity in the F/F_0_ signal within the cytosol. The model line scan profiles below (Fig 5D) show the changing intensity across the middle of the transverse plane (along the yellow line), with zero fluorescence within mitochondrial regions. An experimentally measured confocal point spread function (PSF) was then convolved with the model images to simulate confocal visualizations of the signals for each RyR cluster distribution. The pixel resolution of the model images was 0.02 μm in X and Y and therefore, the images were also scaled down 10 times to match the typical pixel resolution with which line-scans are acquired. The images in Fig 5C and corresponding simulated confocal line profiles in Fig 5D show that the drop in intensity within mitochondrial regions is less apparent in the confocal-resolution line scans.

Fig 5E is an estimate of the density of RyR clusters per unit volume, within a 1.0 μm radius spherical neighborhood, across the length of the line. Comparison with the confocal line plots in Fig 5D shows that high intensities in a confocal line scan from a RyR cluster distribution correlate with a higher density of RyR clusters in the local neighborhood. Therefore we believe that the heterogeneity during the rising phase of the [Ca^2+^]_i_ transient is a composite of non-ECC structural components, such as the mitochondria, as well as the number of Ca^2+^ release sites in the vicinity of the line.

In addition, it is worth emphasizing that the variations to the RyR cluster distribution in the three simulations are within the experimentally observed and statistically quantified variations in our RyR cluster distribution data (as described in S1 Text). As such, Fig 5C and 5D show that experimentally observed variations to structural organization of RyR clusters could give rise to variations in heterogeneity at line scan resolution.

### Direct measurement of the neighborhood of clusters that contribute to a missing line scan “spark”

Several animal models of heart failure exhibit spatial heterogeneities in line scan profiles. Heart-failure is known to cause defects in t-tubule organization that consequently affect the association of RyR clusters with the t-tubules that is necessary for effective CICR [19,24,42–44]. The dissociation of RyR clusters from the t-tubules appears as a drop in fluorescence intensity along the line scan, suggesting that RyR clusters have not been activated by the action potential [19,42]. Using our spatially-realistic and biophysics-based model, we set out to directly investigate the number of RyR clusters and the area of their neighborhood that must be un-activated in order to reproduce a regional drop in fluorescence as in a disease state.

**Fig 6:**
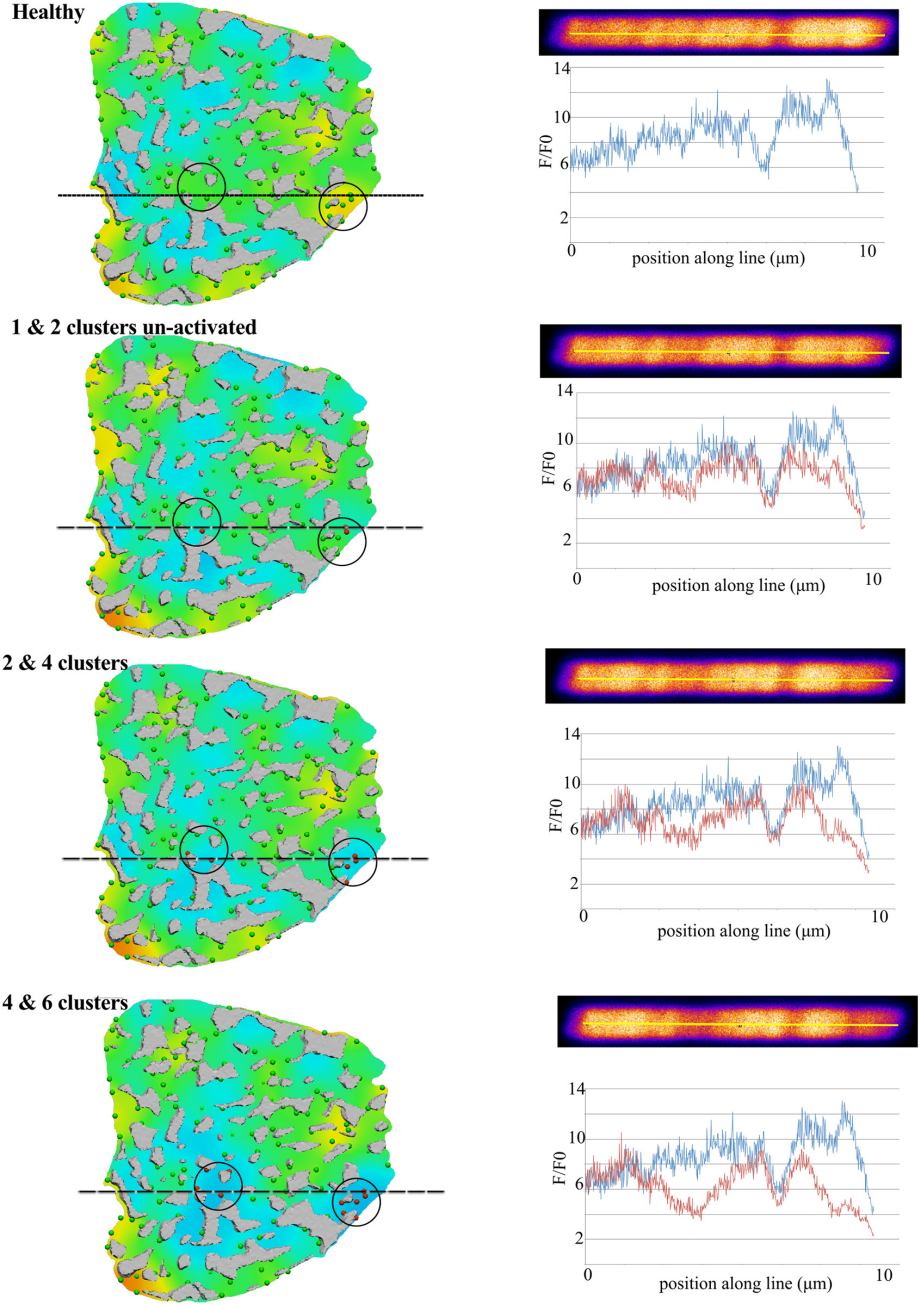
4–6 orphaned RyR clusters contribute to a missing “spark” in line scans. View of [F4Ca]_i_ in the default, “healthy” model and “diseased” models (rows 2 to 4) with increasing number of un-activated RyR clusters; regions of interest are encircled in black and subsequent images show the un-activated RyR clusters in these regions, highlighted in red. The line plot for the default model is shown in all the plots for easy comparison with the line plots from the increasingly “diseased” RyR cluster distributions. All images were acquired at t = 30 ms.

Fig 6 shows the in-plane fluorescence (left), the simulated transverse line scan images (right, upper panel) and the line scan profile (right, lower panel) as we increased the number of RyR clusters that remained un-activated through the rising phase of the [Ca^2+^]_i_ transient (shown as red spheres in the left column; regions under investigation are encircled in black). The top row is the default, healthy model and the subsequent rows show the drop in fluorescence in the local regions where RyR clusters were prevented from releasing Ca^2+^. Differences in the line scan profile are observable when 1 RyR cluster is un-activated (second row), but the symptom of “disease” on the line scan image is only observable when the local fluorescence remains at the basal value (i.e., with F/F_0_ ≈ 1.0). This corresponds to 4 or 6 RyR clusters remaining un-activated as shown in the last row of Fig 6. In addition, because the spatial model is consistent with structural microscopy data, we can infer that the 4 to 6 RyR clusters span a 1.29 μm diameter neighborhood.

### Global [Ca^2+^]_i_ is robust to significant cross-species differences in RyR cluster distribution

We have demonstrated how our method for generating realistic distributions of RyR clusters enables us to examine heterogeneities in [Ca^2+^]_i_ due to the RyR cluster distribution. Fig 4 shows that we can also simulate variations in the RyR cluster distribution, which are equivalent to variations seen in structural imaging data. These variations affected the spatial profile of [Ca^2+^]_i_, but had no effect on the range of [Ca^2+^]_i_ in the cytosol. As such, [Ca^2+^]_i_ is robust to variations in RyR cluster organization observed within our experimental data of RyR cluster distributions in adult rat ventricular myocytes.

A previous comparison of RyR cluster distributions in rat and human cardiomyocytes [9,23] found that RyR clusters were less densely populated (40% drop) and more spread out (mean nearest-neighborhood distance ≈ 0.79 μm) in human cardiomyocytes. We hypothesized that this drop in the density of clusters would give rise to a more heterogeneous distribution of [Ca^2+^]_i_.

We simulated a human-equivalent distribution of RyR clusters (87 clusters with a mean nearest-neighborhood distance of 0.79 μm) within the electron-tomogram template distribution of myofibrils and mitochondria from the Wistar rat myocyte; the comparative studies between rat and human myocytes showed that the volume fraction of mitochondria and myofibrils were not different between the two species [9].

Ca^2+^ release and diffusion was simulated on this human-type myocyte to explore [Ca^2+^]_i_ heterogeneity. Ca^2+^ release current from each of the 87 clusters was increased to 2.8 pA to compensate for the drop in the number of clusters in the model and maintain a whole-cell average [Ca^2+^]_i_ of 1 μM that is also measured in human myocytes [45]. This compensation in RyR [Ca^2+^] release is also consistent with recent experimental findings that the cardiac Ca^2+^ store and release systems maintain long-term balance in Ca^2+^ through alterations to SR Ca^2+^ content and the strength and duration of Ca^2+^ release from RyR clusters [46,47].

**Fig 7:**
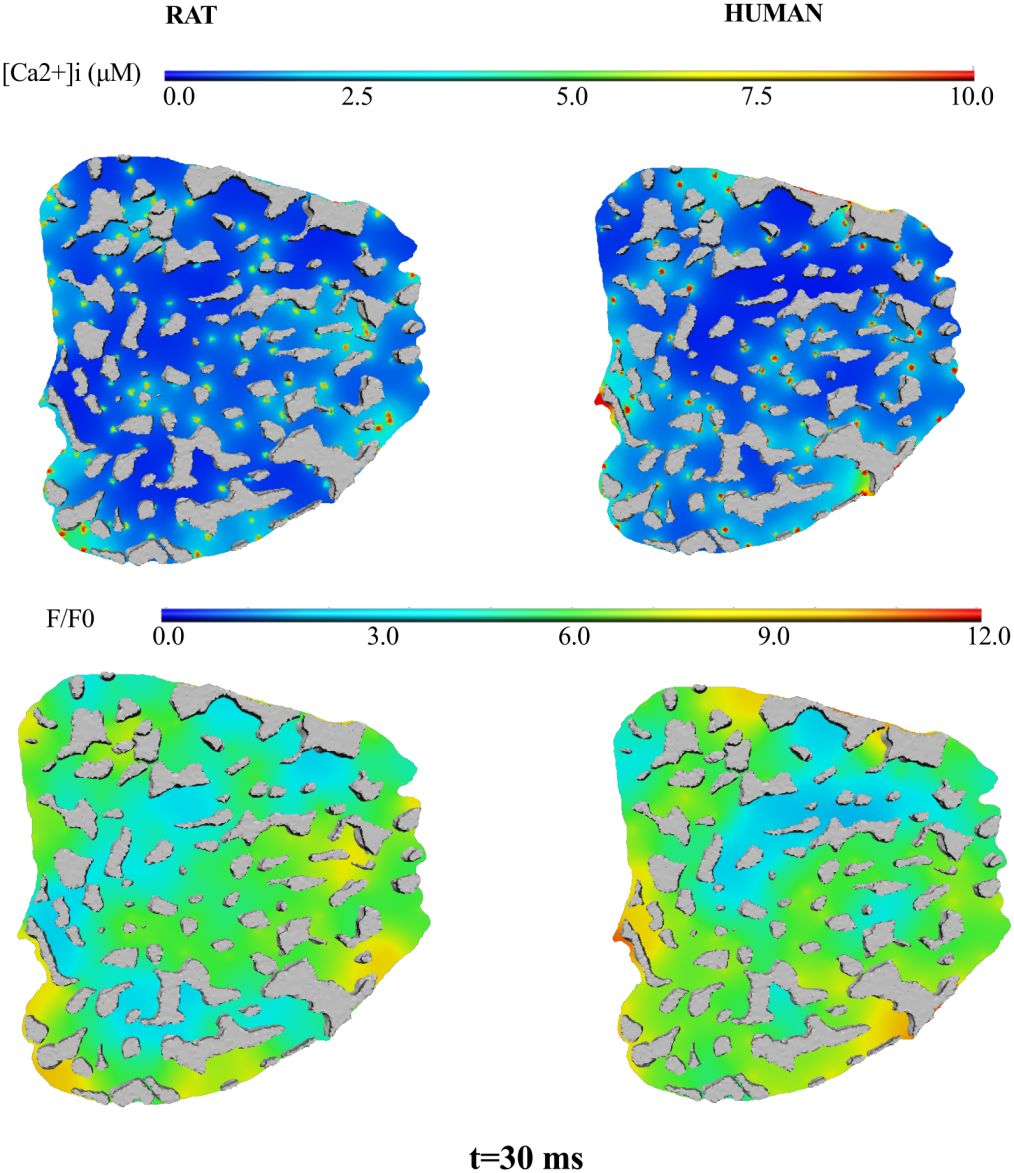
[Ca^2+^]_i_ is robust to alterations in RyR cluster distribution. [Ca^2+^]_i_ (top panel) and [F4Ca]_i_ (bottom panel) are shown at t = 30 ms, at the z-disc transverse plane. Results on the left column were generated from a simulation using RyR cluster distribution properties of the rat. Results on the right column were generated from a simulation using RyR cluster distribution properties from human myocyte measurements.

Fig 7 shows a comparison of the spatial distribution of free [Ca^2+^]_i_ (top panel) and Fluo-4-bound Ca^2+^, [F4Ca]_i_ (bottom panel) in the rat (left column) and human (right column) models. The range of [F4Ca]_i_ and [Ca^2+^]_i_ are the same in both species and although the locations of local hot-spots may be in different regions in the two models, the spatial profiles are similar. This analysis illustrates the robustness of [Ca^2+^]_i_ dynamics to a significant change in RyR cluster distribution properties.

## Discussion

We have presented a novel hybrid-scale spatial model of the organization of clusters of ryanodine receptors around cardiac cell myofibrils and mitochondria. The model is spatially realistic, owing to a novel computational fusion of 3D confocal microscopy data of RyR clusters with a 3D electron tomogram of myofibrils and mitochondria acquired from tissue samples of Wistar adult rat ventricular myocytes. This model was used to examine the spatio-temporal dynamics of [Ca^2+^]_i_ at a much higher resolution than was previously possible and has given several important insights on the role that structural organization can play in affecting [Ca^2+^]_i_ in health and disease. We discuss some of the key advances and findings from this study in the following sections.

### Computational fusion of different experimental datasets

Several microscopy methods are required to visualize different aspects of cardiac cell structure. Confocal and super-resolution methods give the advantages of high-contrast (due to immunostaining) and large fields of view [4,7,14]. However, potential crosstalk between different antibody label signals limits the number of proteins and organelles that can be visualized from one cell. In contrast, 3D electron tomography and more recently developed serial-block-face imaging methods provide higher resolution, but relatively poor contrast [8,11]. As such, the different microscopy methods complement each other and could be used in conjunction to generate a unified view of the cell.

**Fig 8:**
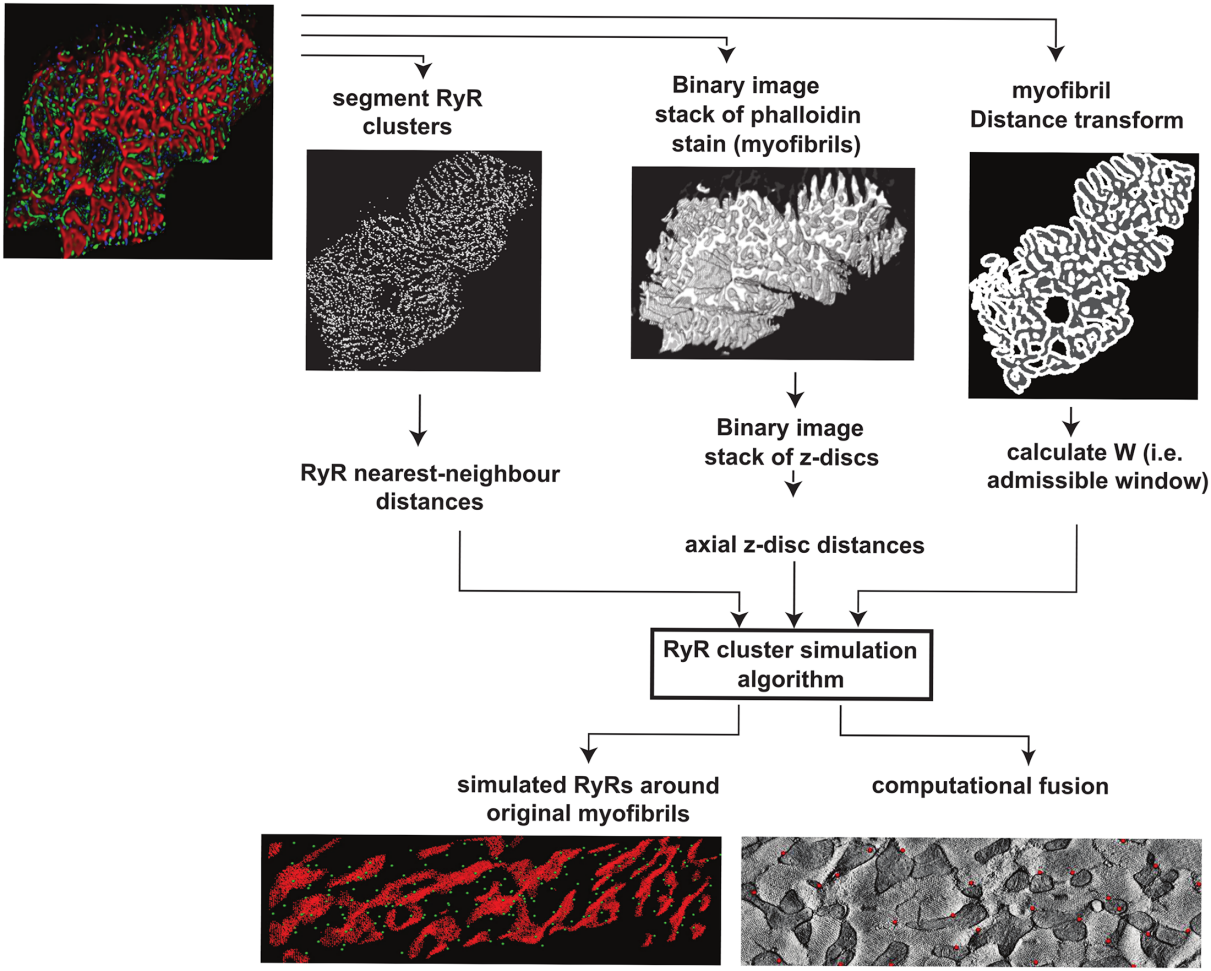
RyR cluster simulation algorithm workflow. (1) segment RyR clusters from original data; (2) calculate the nearest-neighborhood distances for the RyR clusters; (3) Identify Z-disc planes from RyR cluster data; (4) Convert phalloidin stack into binary image stack; (5) Identify Z-plane positions on the binary myofibrils stack from (4) and set rest of the planes to background colour; (6) Calculate background distance transform for z-disc stack and decompose to radial and axial components; (7) Determine W, window of voxels available for RyR cluster simulation; (8) Use W, nearest-neighborhood distances and axial and radial z-disc distance statistics as input for RyR cluster simulation algorithm.

Our method (detailed in S1 Text and Fig 8) of analyzing the spatial relationships between ion channels and the surrounding cellular machinery enables us to generate a unified view of different cardiac cell components from different microscopy methods. Although correlated microscopy methods are in development [48], our method also provides an added benefit of capturing the variations in the organization that are observed in experimental data, both among healthy populations and across different disease states. As such, computational analysis of spatial relationships between different components of the cell is an important aspect of cardiac structure analysis. Our algorithm for RyR cluster simulations can be applied to generate computer models of RyR clusters and contractile machinery with other templates of myofibril and mitochondrial organization taken from EM tomography as well as serial-block-face imaging and confocal imaging technologies. The approach of modeling RyR clusters as point processes could be translated not only to other ion channels in the cardiac cell (e.g., L-type Ca^2+^ channels and sodium-calcium exchangers), but also to ion channels in all cell types in general.

### Realistic, irregular distributions of RyR clusters can affect local [Ca^2+^]_i_

Our spatially-accurate model enabled us to explore how structural components of the cell contribute to spatial non-uniformities observed in the rising [Ca^2+^]_i_ transient [41]. In our analysis of distributions of RyR clusters, we found that RyR clusters aggregate into groups and can therefore give rise to local elevations in [Ca^2+^]_i_ (see Figs 2, 3 and 4). These heterogeneities in RyR cluster distribution could also give rise to spatial heterogeneities in line scan images of the [Ca^2+^]_i_ transient (see Fig 5). More idealized models of RyR cluster distributions as a regular grid of ion channels [32,35] would not be able to capture these effects.

We also examined the effect of heterogeneous distributions of mitochondria—which act as barriers—on spatiotemporal [Ca^2+^]_i_ dynamics. Fig 3 shows that assigning mitochondria to have the same diffusive properties as the cytosol reduces the occurrence of local elevations in [Ca^2+^]_i_. We did not incorporate the Ca^2+^ buffering capacity of mitochondria in our simulations. Recent studies indicate that the experimentally measured mitochondrial Ca^2+^ uptake rates are at least two orders of magnitude lower than those required to significantly and rapidly affect cytosolic calcium levels under physiological conditions [39,40]. Therefore, incorporating a component of mitochondrial Ca^2+^ uptake mechanism is unlikely to alter the concentration gradients (and dampen a 3- to 100-fold heterogeneity in cytoplasmic Ca^2+^) that is predicted when modelling mitochondria as passive barriers to Ca^2+^ diffusion.

We tested the model’s sensitivity to buffering by incorporating calmodulin and ATP. S6A and S6B Fig show that the heterogeneity in the F/F_0_ signal at the end of the 30 ms simulation drops with CaM and ATP buffering. A look at the distribution of Calmodulin-bound Ca^2+^ (bottom right) shows that the heterogeneity introduced by the structural organization now manifests in the buffered CaM-Ca distribution.

We also investigated the sensitivity of the observed [Ca^2+^]_i_ heterogeneity to the biophysical detail in RyR gating. As detailed in S2 Text, we implemented a previously published two-state Markov model of RyR gating to account for: Ca^2+^ dependent RyR gating; sensitivity of RyR gating to Ca^2+^ in the junctional sarcoplasmic reticulum (JSR); and stochastic behavior of individual ion-channel gating [49,50]. Intracellular Mg^2+^ is also known to affect RyR gating kinetics. Experimental and modeling studies such as [51–53] have demonstrated the inhibitory effect of Mg^2+^ in lipid bilayer studies by performing RyR gating experiments at different concentrations of Mg^2+^. The parameters of our chosen model account for physiological levels of Mg^2+^ and recapitulate several of the experimentally observed spark properties under physiological conditions.

Each of the clusters in our whole-cell model was assumed to contain 50 individual RyRs. A deterministic approximation of the two-state Markov model was used to simulate average behavior of the cluster of RyRs [54]. Each cluster was triggered to release Ca^2+^ from the JSR into the dyadic space by raising the mean open probability from 0 to 0.02, which is equivalent to a single RyR channel opening due to the L-type Ca^2+^ stimulus. S6C Fig shows that our observations of heterogeneity in the Ca^2+^ transient and Calmodulin-bound Ca^2+^ still hold.

These results, together with Figs 2, 3 and 4 clearly confirm that the spatial organization of RyR clusters and mitochondria introduce heterogeneity to the Ca^2+^ transient. Previous studies typically assume that the rising [Ca^2+^]_i_ transient is homogeneous under control or normal physiological conditions. We have for the first time shown with a structurally realistic model, how the “normal” structural organization of RyR clusters alone can introduce spatial heterogeneities in the rising Ca^2+^ transient.

### Line scan “sparks” involve 1.29 μm neighborhood of RyR clusters

Our simulations of missing sparks on the confocal line scan image (see Fig 6 and S7 Fig) demonstrated that a 1.29 μm diameter neighborhood of RyR clusters—corresponding to 4 to 6 RyR clusters—must remain un-activated to produce a line scan image with a local drop in fluorescence. This shows the power of a structurally realistic model in enabling the exploration of the effects of systematic modifications to structure on the local control of [Ca^2+^]_i_ dynamics. A previous experimental analysis [37] of the relationship between single L-type Ca^2+^ release channels and RyR channels had shown that a single L-type channel can trigger 4 to 6 RyR channels; here we have quantified the contribution of RyR release at a higher spatial scale (in several cluster groups of RyRs, each cluster containing many single RyRs [55]). Our result is limited to the rising phase of the action potential evoked transient. Simulations involving longer than 30 ms duration would need to include proteins involved in Ca^2+^ reuptake to simulate the observed recovery of spatial uniformity [42]. As such, our current observation is valid for the initial phase of the transient.

### [Ca^2+^]_i_ transient is robust to cross-species RyR cluster distributions

Our comparison (see Fig 7) of the spatiotemporal dynamics of [Ca^2+^]_i_ between a simulated rat and a simulated human cardiomyocyte—defined by the different RyR cluster distributions measured in literature [9,23]–suggests that the Ca^2+^ diffusion mechanism and the organization of myofibrils and mitochondria provide significant robustness to [Ca^2+^]_i_ dynamics. In particular, a 40% drop in density of RyR clusters has virtually no effect on the spatial profile of [Ca^2+^]_i_. This comparison of the cluster distribution of human versus rat RyR is an important example of how parameterization of structure enables systematic analysis of the sensitivity of cardiac function to specific aspects of cell structure. Such analysis is not possible when generating a model from a single dataset.

Our simulations assumed that all RyR clusters were activated by the action potential. The impact of the difference in t-tubule organization between human and rat cardiomyocytes has not been thoroughly investigated in this study. However, the observation that t-tubules in the human myocyte are more coarsely arranged than in the rat myocyte is consistent with the more dispersed, and less dense RyR cluster parameters that we used to simulate human myocytes in this study [9]. Previous structural studies have also observed a small proportion of RyR clusters that were not associated with t-tubules and therefore may not be activated by the action potential [56]. However, these were largely in the periphery, and we anticipate that this sub-population will have limited effect on the current computational observation. In addition, although our “best-case” scenario of all RyR clusters being activated may not be completely realistic, varying strengths of release from each RyR cluster could compensate for any local loss of RyR cluster Ca^2+^, thus adding more robustness to the ECC system.

### Limitations and future directions

Our current analysis focused on the possible role that realistic distributions of RyR clusters, mitochondria and myofibrils could play in the spatio-temporal dynamics of [Ca^2+^]_i_. The ability to model RyR cluster distributions enabled us to explore an aspect of cardiac cell structure as a model parameter within experimental bounds. The degree of influence that this structural parameter may have on cell biophysics was systematically investigated with simplified and biophysically detailed models of [Ca^2+^]_i_ dynamics and buffering.

Inherently, electron tomograms are low contrast, which can hamper the identification of boundaries between myofibrils. Being aware of the fact that myofibrils can bundle together and also twist through the cell volume [57], we segmented myofibrils as precisely as possible given the quality of our image dataset. More careful characterization of myofibril and mitochondrial regions in tomograms and new serial-block-face imaged samples [11] will enable us to improve on the current segmentations. Nevertheless, it is worth noting that we anticipate minor refinements to the current findings because our simulation of the average cell [Ca^2+^]_i_ transients are consistent with experimental records of Ca^2+^ in cardiomyocytes.

There are many more structural components in the cell, one of which is the t-tubule network that traverses between the myofibrillar and mitochondrial regions. As the field of view in the tomogram used for this study is small, only a few t-tubule openings could be observed. A more complete geometric model would incorporate t-tubules and will potentially introduce more heterogeneity as already observed in previous simulation studies that examined Ca^2+^ release and diffusion around realistic t-tubule geometries [12,15]. Furthermore, more high-throughput methods for imaging cardiac cell myofibrils and mitochondria [11] will enable us to extend the current half-sarcomere model to encapsulate realistic organizations of the myofibrils and mitochondria in the longitudinal direction as well.

With the advent of super-resolution microscopy, higher-resolution information on the distribution of RyRs is coming to light [4,7,55]. Finer details on the spread of individual RyR channels within clusters of RyRs are now being investigated. As such, incorporating this new information will refine our current findings. We aim to apply our method for modeling RyR cluster distributions to this new data and examine how these different spatial scales of information integrate to give rise to the whole cell transient.

A full ECC cycle would incorporate other sources and sinks of Ca^2+^- particularly sarcoplasmic reticulum Ca^2+^ (SERCA) pumps. It is our medium-term goal to systematically analyze the spatial distribution of other key ECC proteins and to incorporate them into our hybrid-scale spatial model of the cardiac cell. As we have demonstrated in this study, the ability to characterize the spatial arrangement of structural components using statistical models enables us to examine structure as a model variable that current image-derived models cannot do. As cells undergo structural remodelling through many signaling mechanisms, parameterization of spatial relationships between structural components is an important step towards quantifying the dynamic relationship between structure and function in both health and disease.

## Materials and Methods

### Ethics statement

All animal procedures followed guidelines approved by the University of Auckland Animal Ethics Committee (for animal procedures conducted in Auckland, Application Number R826) and the University of California San Diego Institutional Animal Care and Use Committee (for animal procedures conducted in UCSD, Protocol SO6215).

### Experimental procedures

#### Confocal microscopy for RyR cluster distribution analysis across cells

Analysis of RyR cluster distributions and the validation of the method for fusing confocal-level protein distributions with electron tomography data were conducted using four cells (numbered in Fig 1A) from confocal images of left ventricular cardiomyocytes from a healthy adult male Wistar rat.

We used well-established tissue fixation and immuno-labeling protocols to prepare tissue samples for confocal imaging [14,56]. Clusters of RyR2 (shown in green fluorescence in Fig 1B) were labeled using mouse monoclonal anti-RyR2 antibodies (clone C3-33) purchased from Affinity Bioreagents (Golden, CO, USA). Rabbit monoclonal anti-plectin antibodies (E398P, Abcam) were used to mark z-disk locations along the length of the cells. The myofibrils (shown in red fluorescence in Fig 1B) of the myocytes were labeled using Alexa Fluor 647 phalloidin (Invitrogen). Flourescence images were recorded with a Zeiss LSM710 laser scanning confocal microscope using a Zeiss x63 NA 1.4 oil-immersion objective. Stacks of confocal sections (0.2 μm spacing between adjacent sections; 0.07 μm spacing in X and Y) were acquired for offline three-dimensional data processing. To enhance the data for further analysis, iterative constrained deconvolution was performed using a Richardson-Lucy algorithm similar as previously described [58] using custom routines in the PYthon-Microscopy Environment (PYME; code.google.com/p/python-microscopy).

RyR clusters were detected using an object finder algorithm using multiple passes of a bandpass filter detector. After each pass, detected clusters were removed from the image data set to enable the detection of fainter clusters in the next pass, broadly similar to the method described by Soeller et al [23]. The centroid locations of detected puncta were determined and inserted into the list of detected RyR clusters. The list of RyR cluster locations (displayed for each cell in S1 Fig) was then used in the point statistics analyses described in S1 Text. All detection routines were implemented with a graphical user interface in PYME.

#### Electron tomography for high-resolution images of the organization of myofibrils and mitochondria

We collected a 3D electron microscopy image stack (tomogram) of a left ventricular myocyte to visualize the anatomical distribution of the myofibrils and mitochondria in the cell cross-section (see Fig 1C). These datasets were extracted from an adult male Wistar rat heart that was prepared separately from those collected for confocal microscopy, following tissue preparation and staining protocols similar to those published in the literature [8]. 300 nanometer sections were acquired and stained with 2% aqueous UA and Sato’s lead stain. The samples were stained with 15 nanometer colloidal gold for tomographic reconstruction. A tilt series from -60 to +60 was acquired on a 300kV FEI system at 4K magnification. The resulting tomogram had a spatial resolution of 35 nm in all three dimensions. The physical dimensions of the collected cell volume spanned the entire cross-section of a cell within the tissue block (94.8 μm^2^ in cross-sectional area) and 240 nm in the longitudinal dimension. The thickness is limited to 240 nm because of the limitation that transmission electron microscope images lose contrast with increasing tissue sample thickness.

The 3D image stack was manually segmented for the sarcolemma and the boundaries of mitochondria and myofibrils (see Fig 1C and S2A and S2B Fig) using the open-source electron microscopy image processing software, IMOD [59]. There are many other organelle and membrane components visible in the tomograms (including t-tubule segments and portions of sarcoplasmic reticulum), but only the myofibrils and mitochondria were segmented for the current investigation. Clumped mitochondria that we observed within the tomogram were deemed as one large functional mitochondrial unit with no barriers between them due to mitochondrial fusion [60,61]. The myofibril and mitochondrial segmentations were saved as separate binary image stacks for finite element mesh generation and RyR cluster simulations as detailed below.

### Computational mesh generation and biophysical simulation of [Ca^2+^]_i_

#### Mesh generation

The binary image stacks of myofibril and mitochondria cover the entire cross-section of a cell, but only span 240 nm as explained above. Using the fact that RyR clusters predominantly localize to the z-disc and the observation that myofibrils follow straight paths over two or three sarcomeres [57], we compensated for the 240 nm depth-limitation by extending the segmented image stacks symmetrically on either ends to generate half-sarcomere (0.9 μm) image stacks (see S2C Fig). This approach enabled us to investigate [Ca^2+^]_i_ dynamics in a structurally realistic three-dimensional topology.

The extended geometric stacks were then processed with an open-source MATLAB program, iso2mesh [62], to generate a 3D finite element mesh as shown in S2D Fig. The mesh consists of 222,312 nodes and 1,307,928 linear simplex tetrahedral elements and is described in TetGen [63] format. The elements were grouped as mitochondria and myofibrils—the purely cytosolic region between the myofibrils and mitochondria is a very small fraction of the cell space (see Fig 1D and S2A Fig) and was thus assumed to be negligible in the simulations. The boundary interfaces between mitochondria, myofibrils, and the sarcolemma were labeled in order to facilitate applying boundary conditions in the finite element model. Mitochondria were modeled as Ca^2+^ diffusion barriers and did not act as Ca^2+^ buffers; this assumption was based on recent experimental studies [39,40]. No flux boundary conditions were applied to the sarcolemmal nodes and both extremities of the half-sarcomere, assuming symmetry in Ca^2+^ release and dynamics from surrounding z-discs.

#### [Ca^2+^]_i_ dynamics

[Ca^2+^]_i_ dynamics simulations were conducted on the computational mesh using coupled reaction-diffusion equations for free [Ca^2+^]_i_, free Fluo-4-AM, [F4]_i_ (a Ca^2+^- sensitive fluorescent indicator) and Fluo-4-bound-Ca^2+^ ([F4Ca]_i_). The role of myofibrillar Troponin C as an immobile buffer of Ca^2+^ was also incorporated. Mesh nodes that fell within a 200 nm diameter spherical neighborhood of simulated RyR cluster coordinates (method described below and in S1 Text) were assigned as RyR release sites in order to represent Ca^2+^ release from the dyad into the cytosolic space as in [29]. Additional details of the equations used for the simulations are provided in S2 Text. Parameters for the rate constants, diffusivities and initial conditions are provided in Table 1.

All finite element simulations were conducted using the open-source computational modeling platform OpenCMISS [64]. The reaction terms and buffering parameters were defined using CellML and coupled to the reaction-diffusion PDE using the Strang operator splitting method [65].

### Computational fusion of confocal-scale RyR cluster distributions with electron tomography data

We developed a novel algorithm—using spatial point process statistics techniques [16,17]—that generates realistic confocal-scale RyR cluster distributions around nanometer-resolution 3D image stacks of myofibrils and mitochondria organization acquired using 3D electron tomography.

#### Spatial statistics fundamentals

We model the locations of the RyR clusters within the cell as a *point pattern* where each point represents the centroid location of a single RyR cluster. A point pattern has an associated generating mechanism known as a *point process*. Therefore, we use a number of analytic techniques from the spatial statistics field. We provide a brief, conceptual background on the techniques used here; additional detail is available in the literature [16,66,67].

In most instances, point processes can be characterized by their *intensity function λ*(*x*) (physically interpretable as a spatially varying density of points per unit volume) and how the points of the pattern interact (e.g., inter-point repulsion or attraction given by measures such as the nearest-neighborhood distance distribution between points of the point process). The simplest model is the Poisson point process, where point locations are independent of one another.

The constant intensity Poisson process (*λ*(*x*) ≡ *λ*) is said to exhibit “Complete Spatial Randomness” (CSR). In this model, intensity (number of points per unit volume) does not vary spatially and there is no interaction between the points of the pattern. This is often the initial model fit when analyzing point patterns.

A number of summary statistics exist for investigating departures from CSR. The four measures we use in this paper are:

- The *F* function, *F*(*r*), is the probability that the shortest distance, d(u,**X**), from an arbitrary point *u* to a point of the point pattern ***X*** is less than or equal to *r: $d(u,X)\;=\;min\{\left|\left|u-x_{i}\right|\right|:u\in W,\;x_{i}\in X.$* It is typically physically interpreted as a measure of empty space in the point pattern.
- The *G* function, *G*(*r*), is the probability that the shortest distance, d(u,**X**{u}), from an arbitrary point *u* of the point pattern ***X*** to another point of *X* is less than or equal to *r* and is the cumulative nearest-neighborhood distance measure: *G*(*r*) = *P*{*d*(*u*,***X***{*u*}) ≤ *r*: *u* ∊ ***X***
- The K-function [68] is derived from the pairwise distances between all the points in the process, defined by: $s_{ij}\;=\;\left|\left|x_{i}-x_{j}\right|\right|$. The K-function represents the expected number of other points that fall within a sphere of radius, r, of an arbitrary point of the process, u: $K(r)\;=\;\frac{1}{\lambda }E\left[N\left(X\cap B(u,r)\{u\right\}\right):\;u\in X]$, where *λ* is the average number of points per unit volume, B(u,r) is a sphere centered on u with radius r, and E[] is the notation for the expected (or mean) number of points.
- The pair correlation function (PCF) for a stationary point process is defined as as: $g(r)\;=\;\frac{K'(r)}{2\pi r}$. The numerator is the derivative of the K-function of the point process in question and the denominator is the derivative of the K-function of the Poisson point process. Thus, the PCF measures the correlation in the point process relative to the theoretical Poisson point process (in which there is no correlation in the locations between points).

#### Monte-Carlo simulations and envelope-based hypothesis testing to compare experimentally observed and model distributions of RyR clusters

Using many instances of spatial point patterns that are simulated from a proposed point process model (termed Monte-Carlo simulations), one can then determine the maximum and minimum possible values of the above spatial measures out of all the simulated patterns for each radius neighborhood, r_d_. These bounds provide “envelopes” that can be used to determine whether there is evidence against the hypothesis that an observed point pattern could have been generated by the proposed point process model 16,66,68,69].

As mentioned above, we performed an initial hypothesis test that the RyR clusters within the rectangular windows in S1 Fig can be described as completely spatially random. S8 Fig shows envelopes generated for F, G, K and PCF by picking their maximum and minimum values from a set of 99 simulations generated using the complete spatial randomness process model. The model simulations were generated in an attempt to create RyR cluster distributions that had similar spatial statistical characteristics to the observed distribution within the rectangular window in the first cell in S1 Fig. The black curves in each envelope plot in S8 Fig represent the estimated values of F, G, K and PCF for the observed RyR cluster distribution in cell 1. An examination of the G, K and PCF function plots (S1B, S1C and S1D Fig, respectively) shows that the measured functions from the experimental data deviate away from the envelope of values for that function if the cluster distribution was assumed to be completely spatially random. These envelope plots are therefore evidence against the null hypothesis that the RyR cluster distribution can be simulated by completely spatially random process. It is this method of hypothesis testing that we used in the subsequent analysis to determine a model and algorithm to simulate RyR cluster distributions that captured the statistical characteristics of observed RyR cluster distributions. S8 Fig also demonstrates the importance of examining all four spatial metrics (as opposed to one or two of them) because each spatial metric only captures one aspect of the interaction between the points. S8 Fig shows that a model could capture the F metric of an observed spatial point pattern, but might not capture the other three spatial metrics.

#### Algorithm approach to simulate RyR cluster distributions in cardiac cells

Having determined in S8 Fig that a simplistic homogeneous distribution of RyR clusters is insufficient to capture the realistic distributions observed in experiments, we then used an experimental data-driven approach to simulate RyR cluster distributions around myofibrils. Known in general as the *reconstruction algorithm* in spatial statistics theory, the method uses energy minimization techniques to match a target set of statistical measures of an observed point process [16] (see S1 Text for mathematical details). The aim here was to determine the most important statistical measures of the experimental RyR cluster distributions that must be recapitulated by simulated RyR cluster patterns in order to generate realistic distributions. As such, this data-driven approach enables us to immediately identify the key spatial metrics in the experimental data, rather than using a parametric approach that involves fitting parameters of theoretical models of spatial distributions that may not have direct physical interpretations.

The algorithm development involved the following key steps:

1. Analysis of the spatial distribution of RyR clusters in confocal image stacks (see Fig 1A and 1B and S1 Fig) as a spatial point process [16] relative to the myofibril contractile machinery using the above metrics and Monte Carlo approaches.
2. Determining the key spatial point metrics (such as nearest-neighborhood distance or pair-wise distances between RyR clusters) that are required to simulate RyR cluster distributions that are statistically equivalent to the experimentally observed spatial distribution.
3. Simulating RyR cluster distributions on a target image stack of segmented myofibrils and mitochondria—such as the half-sarcomere image stack in S2C Fig—using the identified spatial metrics.

#### Computer implementation of RyR cluster model simulation

Our numerical implementation of the RyR cluster simulation algorithm is provided in a pictorial flow diagram in Fig 8. The implementation works on voxels and the image domain to make full use of the imaging data and image processing tools for high-throughput statistical analysis and validation of this proposed technique. We iterate the steps as follows:

1. Segment RyR clusters from confocal image stacks (similar to Fig 1B) and extract centroid positions for each cardiac cell. We label the observed spatial point pattern of RyR clusters, ***X***.
2. Calculate the nearest-neighborhood function, G(r), for the observed RyR cluster pattern.
3. Find planes through the cross-section depth with maximal RyR cluster points and identify those planes as the location of the z-discs.
  Due to the inherent limitations of confocal microscopy techniques, the image resolution in the longitudinal direction is much lower than that of the cross-section (as shown in Fig 1B). However, based on previous studies, it is well known that RyR clusters aggregate strongly to the z-discs [14]. Thus, we modified the segmented RyR cluster data such that all segmented RyR clusters were at z-disc planes. In the current simplified model, the z-discs for these cells are assumed to align over the cross-section and the current statistical model does not account for variations in axial aggregation of RyR clusters around these z-discs.
4. Threshold the phalloidin stack to create a 3D binary image with white voxels representing the myofibril regions and the black voxels representing non-myofibril regions.
5. Create a new binary image stack, derived from the stack in step (4), by setting all voxel values to background in all the image slices, except slices that represent z-disc locations identified in step (2).
6. Construct the z-disc distance function (see S9 Fig) as a frequency histogram of RyR clusters at different distance bands away from the z-disc by calculating the background distance transform [70] from the RyR cluster centroids in step (1) to the z-disc stack white voxels of step (5). Note that for this particular study, we set the z-disc axial distance to zero (as explained in step (3)).
7. Determine the window, W, in which the RyR clusters can be simulated by using the reconstruction algorithm as follows: Calculate the distance transform from each background voxel to the myofibrillar voxels in the image stack in step (4); the available voxels in which RyR clusters are observed and, consequently, can be simulated is determined as the voxels within a 0.7 μm radial distance of the nearest myofibril boundary. The radial distance from RyR cluster centroids to myofibrils were also extracted using this distance transform.

From this workflow, we could determine the G statistic, axial distances, and radial distances for the observed dataset. These measures were then used in the reconstruction algorithm to simulate a new RyR cluster pattern in the same cell inside W, or in a new cell image stack for which W had been calculated by following step (6).

Further details of the RyR cluster distribution analysis and algorithm development are provided in S1 Text. Readers are guided to the results section for key points of this method that enable the generation of realistic RyR cluster distributions on electron tomography data of myofibrils and mitochondria.

123 RyR cluster positions (with a nearest-neighbour distribution that was consistent with the experimentally observed distribution in the confocal datasets we collected (Fig 1A) and consistent with previous studies [8,9,23]) were simulated on the half-sarcomere binary image stacks depicted in S2C Fig. The simulated centroid positions (see Fig 1E) were assigned to mesh nodes within a 200 nm spherical neighborhood of each position in order to represent the SR Ca^2+^ release sites.

### Resource repository

We have created an online repository at https://github.com/vraj004/RyR-simulator, containing the source code for the RyR cluster simulation algorithm and necessary input files for simulating RyR cluster distributions on the confocal and electron-tomogram derived geometries. We encourage readers to use the cluster simulation algorithm for fusing with their own image data. We have also created an online repository at https://github.com/vraj004/cardiac_ecc, which contains the files defining the tetrahedral mesh depicted in Fig 1D and 1F. These files can be imported into any computational program that recognizes TetGen formats. The TetGen software also allows for re-export of TetGen files to other commercially recognizable formats such as stl (stereolithography) for easy visualization [62]. The repository also contains all the necessary input files and codes to conduct the [Ca2+]_i_ simulation studies presented in this paper. CellML files defining the Ca^2+^ release profile from each RyR cluster have also been provided (see S3 Fig).

## Supporting Information

S1 Fig RyR cluster distribution study dataset. (A-D) Four cells from the confocal image stack in Fig 1A that were used to study statistical properties of RyR cluster distributions. immuno-labelling of RyR clusters are in green and myofibrils are in red; Middle column shows results of RyR cluster segmentation algorithm; right column shows the rectangular windows (yellow box) in each cell within which the RyR cluster distributions were analyzed; myofibrils shown in grayscale.
(EPS)

S2 Fig Computational mesh generation, from electron tomogram to half-sarcomere. (A): A single slice from the cell electron tomogram showing segmentation of myofibrils, mitochondria and cell boundary. (B): a 3D view of the segmented 240 nm section of the cardiomyocyte. (C): the half-sarcomere extension of the segmented cell in (B); mitochondrial regions are shown in green and myofibril regions are shown in red. (D): final computational mesh with the middle tomogram image slice showing cytosolic region (red mesh lines) and mitochondrial region (gray).
(EPS)

S3 Fig RyR cluster Ca^2+^ release profile. (A) Example time course of Ca^2+^ release from each RyR cluster, with release trigger at t = 0.5 ms. Each RyR cluster was triggered for release with a stochastic variation as reported in literature. (B) Typical Ca^2+^ F/F_0_ spark profile, with full width half max (FWHM) approximately 1 μm. (C) We have also added a sample of sparks in a line scan to illustrate the morphology of the typical sparks in these simulations.
(EPS)

S4 Fig Spatial heterogeneity in Ca^2+^ is independent of the pattern of trigger delay in RyR cluster Ca^2+^ release. With a given RyR cluster distribution, four different triggering patterns were applied and executed to examine the effect of variations in stochastic trigger release on [Ca^2+^]_i_. Top plot shows rise in F/F_0_ signal in the bulk cytosol; the four panels below show the F/F_0_ signal at t = 30 ms for the four different patterns of RyR cluster triggering. Colour coding of panel images below corresponds with line colours in bulk F/F_0_ signal above. Tausim-2 and Tausim-3 had the same time course, thus Tausim-3 is plotted with blue crosses over the red curve representing Tausim-2.
(EPS)

S5 Fig Fluo4 affects spatio-temporal dynamics of [Ca^2+^]_i_. Top curve shows time course of [Ca^2+^]_i_ with corresponding 30 ms snapshot at the bottom when no Fluo-4 was present in the model.
(EPS)

S6 Fig Sensitivity of Ca2+ heterogeneity to Calmodulin and ATP Buffering and Ca^2+^ Dependent RyR Gating Kinetics. The top profile (A) shows the distribution of the F/F_0_ signal at t = 30 ms with the default simulation parameters. (B) shows the F/F_0_ signal when freely diffusing Calmodulin and ATP are introduced as buffering species for Ca^2+^. The right-hand figure shows the distribution of calmodulin-bound Ca^2+^, CaMCa. The image shows that although the heterogeneity in the F/F_0_ signal may be reduced with calmodulin and ATP buffering, the heterogeneity of structure now introduces heterogeneity in buffered Ca^2+^. (C) shows the F/F_0_ and CaMCa signals are still heterogeneous when Ca^2+^ dependent RyR gating kinetics was introduced.
(EPS)

S7 Fig 4–6 orphaned RyR clusters contribute to a missing “spark” with a biophysical model of calcium induced calcium release (CICR). Panel (A) shows a comparison of F/F_0_ at t = 30 ms with 4 to 6 RyR clusters un-activated, using the original model of Ca^2+^ release (left, reproduced from Fig 6) and the biophysically based model of CICR (right). Panel (B) shows the line scan profile along the dashed lines in Panel (A); the profile reproduced from Fig 6 is in red and that for the CICR model is in green.
(EPS)

S8 Fig RyR cluster distributions are not completely spatially random. This figure demonstrates the envelope-based hypothesis test to validate the sufficiency of proposed models of RyR cluster distributions. Black curves represent the measured F (top left), G (top right), K (bottom left) and PCF (bottom right) functions from the segmented RyR cluster distribution of cell 1 at the first z-disc. The shaded regions are the envelopes of possible values for each function if the RyR cluster distribution were modeled as completely spatially random (see S1 Text). The measured G, K and PCF curves fall outside of the envelopes, thus providing evidence against the null hypothesis of complete spatial randomness.
(EPS)

S9 Fig Distribution of radial distances from each segmented RyR cluster across the study dataset. (EPS)

S10 Fig Distribution of nearest-neighbour distances from each segmented RyR cluster to the myofibrils across the dataset. (EPS)

S11 Fig The nearest-neighborhood and radial distance distributions and the number of RyR clusters are sufficient to simulate realistic organizations of RyR clusters. The measured F, G, K and PCF curves for cell 1 fit within the envelopes simulated by the proposed RyR cluster simulation parameters (see S1 Text). The hypothesis test on the other three cells produced similar results.
(EPS)

S12 Fig RyR cluster simulations do not depend on the particular organization of myofibrils. The envelopes for G and K show that RyR clusters simulated on Cell 4, using RyR distribution properties from Cell 1 also fit the RyR cluster distribution in Cell 4. The figure is representative of the other combinations of cells in which the same tests were conducted.
(EPS)

S13 Fig Myofibril radial distance distribution is not necessary to simulate realistic RyR cluster distributions. The envelopes in this figure were generated using the nearest-neighborhood distribution properties and the number of clusters, without the myofibril radial distance distribution. The measured curves from the experimental data still fell within the model envelopes.
(EPS)

S14 Fig Nearest-neighborhood distance is essential to capture the experimentally observed cumulative nearest-neighborhood distribution. The envelope for the G-function was generated by only enforcing a minimum distance between RyR cluster centroids that was equivalent to the confocal resolution. The model envelope does not fit the observed G-function.
(EPS)

S1 Movie Movie showing time-lapse of freely diffusing [Ca^2+^]_i_. (MOV)

S2 Movie Movie showing time-lapse of freely diffusing [F4Ca]. (MOV)

S1 Text Contains details on the analysis of RyR cluster distributions and the development of the computational fusion algorithm. (DOCX)

S2 Text Contains details on the biophysical equations used to simulation Ca^2+^ dynamics. (DOCX)

S1 Table RyR cluster distribution characteristics measured in the four cells in S1 Fig. The cross-sectional area and depth are the dimensions of the field of view within the yellow rectangular windows in S1 Fig. Characteristics of the RyR cluster distribution in the four cells were calculated within this field of view. N is the number of clusters within the field of view; ρ is the density of clusters per unit volume of field of view. An explanation of z-disc radial distance can be found in S1 Text.
(DOCX)

S2 Table Variation in density of RyR clusters per unit cross-sectional z-disc area in each z-disc across the four cells; the values in brackets are the absolute number of RyR clusters at each z-disc. (DOCX)

S3 Table Biochemical rate constants, diffusion constants and initial concentrations used for the Ca^2+^ simulations investigating the effects of calmodulin (CaM) and ATP buffering and Ca^2+^ dependent RyR gating. Parameters were obtained from [29,38,49,50].
(DOCX)
