# Supplementary material for: Examination of the Effects of Heterogeneous Organization of RyR Clusters, Myofibrils and Mitochondria on Ca2+ Release Patterns in Cardiomyocytes
Source: PLoS Comput Biol. 2015 Sep 3;11(9):e1004417. doi: 10.1371/journal.pcbi.1004417 (PMC4559435; doi:10.1371/journal.pcbi.1004417)
Supplement: S1 Text — (DOCX) [file pcbi.1004417.s017.docx]

# S1 Text

This file includes Supplementary Text describing the analysis of RyR cluster distributions and the subsequent algorithm that was developed (and validated) that enables the fusion of realistic distributions of RyR clusters onto electron tomograms.

## Supplementary Text

### Ryanodine Receptor Clusters as Spatial Point Patterns

Ryanodine receptors (RyRs) are ion channels that release Ca^2+^ from the sarcoplasmic reticulum pool into the cytoplasm. These ion channels are also heavily clustered near the z-discs of the myofibrils, where they are tightly coupled to specialized sarcolemma called the transverse-axial tubule system [1]. As such, the ryanodine receptors provide the critical connection between the myofibrils, sarcoplasmic reticulum (SR), and transverse-axial tubules at the z-discs for effective cellular contraction. An anatomically realistic model of the distribution of RyR channels should reproduce the relationship between the RyRs and each of these three cardiac subcellular components.

In this paper, we focus our efforts on quantifying the spatial distribution of clusters of RyRs in relation to the myofibrils and z-discs. The resulting spatial distribution models have immediate applications in understanding the critical role that the spatial relationship between the RyR clusters and myofibrils (and z-discs) plays in efficient excitation-contraction coupling. Here, we present details of the experimental data that we used for this analysis and for algorithm development. We provide the specifics of how we used spatial statistics theory to analyze and simulate the stochastic variations in RyR cluster distributions.

### Experimental Data

Figure 1A and S1 Figure show four cells that were sampled from the left ventricle of an adult male Wistar rat to analyze the spatial distribution of RyR clusters; details of the experimental imaging protocols are provided in Materials and Methods. We segmented the centroids of RyR clusters (middle column, S1 Figure) from these immuno-labelled cells and also segmented the cell surface and the regions representing myofibrils (immuno-labelled with phalloidin, which binds to F-actin – a component of thin filaments – in red in Fig. 1 and S1 Figure). We subsequently examined the spatial distribution of the segmented RyR cluster centroids within a smaller rectangular window (last column in S1 Figure) in order to avoid errors due to edge effects in the subsequent statistical analysis.

### Mathematical Details of Reconstruction Algorithm

Let f(X) be one of the statistical measures of the measured spatial distribution (e.g. F, G, or K). In general, there may also be additional numerical measures n_j_(X) specific to the spatial point process that account for *a priori* knowledge of constraints on the spatial point pattern. *A priori* constraints could include additional user-defined statistical measures not captured by the standard statistical measures defined in spatial statistics theory. In our applications, n_j_(X) typically captures restrictions on the proximity of the point process to other structures that may be present in the space. A simulated pattern X_s_,_0_, is generated in a procedure starting with a set number of points, N_X_, which are randomly distributed over the window of observation, W. Iteratively, the measures f(X_s_,_k_) and n_j_(X_s_,_k_) are evaluated for the current pattern X_s_,_k_ and compared to those of the observed point pattern, X, as expressed through an “error energy” function: E(X_s_,_k_)= E_sf_+E_sn_= $\sum_{i=1}^{I} E_{f,i} +\sum_{j=1}^{J} E_{n,j}$where $E_{f,i}^{S} =(f_{i} -f_{i}^{S} )^{2}$ and $E_{n,j}^{S} =(n_{j} -n_{j}^{S} )^{2}$ with the sums over the chosen functional spatial statistics characteristics indexed by i and numerical measures indexed by j. Points in the simulated pattern are then randomly removed and added in a point-wise fashion, recalculating the energy function each time and terminating when successive energies of X_s,k+1_ fall below a defined tolerance, ε (i.e., E(X_s,k+1_) < ε). After determining a set of parameters that were sufficient to generate realistic RyR cluster distributions using the Monte Carlo simulation and envelope testing method described in Materials and Methods, we found that ε = 0.005 μm^2^ was a sufficient termination value.

### Minimal Parameters to Simulate RyR Cluster Patterns in Cardiac Cells

**The spatial statistical parameters, *f(X)*, and numerical measures, *n_j_(X)*, represent critical spatial parameters/relationships that control the distribution of the RyR clusters.**

**As documented in the literature** [2-4]**, ryanodine receptor clusters aggregate around the z-discs of cardiac cells and closely appose the boundaries of the myofibrils. It is therefore important that a simulated model of RyR cluster distributions reflects these structural characteristics in the experimental data. Therefore, we chose the radial myofibrillar and axial z-disc distances of a cluster as critical parameters that must be matched by the RyR cluster simulation using the reconstruction algorithm. We call these parameters the *observed radial myofibrillar distance function* and *observed axial z-disc distance function* and incorporate them into the energy function as numerical measures *n(X)*. With respect to the energy formulation detailed in section 2.2.3, the mean, the standard deviation, and seven quantiles (12.5%, 25%, 37.5%, 50%, 62.5%, 75% and 87.5%) of the observed and simulated radial myofibrillar and axial z-disc distance function distributions (a total of 9 measures each for axial and radial distance functions) were used to calculate the energy component E_n,j_ at each iteration to ensure that the final simulated patterns matched the observed patterns in these parameters.**

**Regarding the spatial statistical parameters, *f(X)*, we hypothesized that the nearest-neighborhood function (G) – between RyR clusters – is sufficient for an anatomically realistic model of RyR cluster distributions** around **myofibrils. This selection was based on experimental observations that RyR clusters can be triggered by local increases in calcium from neighboring clusters as well as the need for the released calcium to uniformly trigger myofibril contraction across the cell cross-section. Recapitulating the statistical properties of the experimentally measured nearest-neighborhood distance distribution could ensure that these physiological observations are met. Nine measures similar to those for the z-disc distance distribution functions were used to calculate the energy function component E_f,i_ for the nearest-neighborhood distance at each iteration of the reconstruction algorithm as well.**

**Owing to the spatial resolution of confocal microscopes, we observed in all four cell-datasets that the RyR clusters appeared a maximum of 0.7 μm radius away from the myofibrils. Thus, we restricted the possible region within the cell volume from which the points for the simulated RyR cluster pattern, X_s_, can be chosen to be within a 0.7 μm radius of the myofibril boundaries.**

Finally, a critical parameter when characterizing and simulating point patterns is the number of points, N, in the point pattern. Table S1 shows the number of RyR clusters observed in each of the four cells (over a volume of approximately 4 sarcomeres). The table shows that although N may vary between the cells, when normalized by the volume of the field of view, the resulting density, ρ, is consistent across the cells. For the purposes of the simulations on the four cells, we used the observed value of N corresponding to the cell for which the RyR cluster distribution was simulated. The number of clusters at each z-disc was also found to be consistent across the cells (see Table S2). Therefore, when simulating RyR clusters on one z-disc of the electron tomogram template of myofibrils and mitochondria (shown in Fig. 1C and S2 Figure), we used the average number of clusters per unit cross-sectional area of z-disc (1.3 clusters/μm^2^, based on Table S2) to determine the appropriate number of clusters for the model; see the Results section of the main manuscript for further details.

**The following sections show how we tested if this combination of one spatial statistics function and two numerical measures is sufficient to model the RyR cluster distributions observed in our experimental data.**

### Results

### RyR Cluster Distribution Characteristics Across Cells

Table S1 and Table S2 summarize the characteristics of the RyR cluster distributions across the four cells. The nearest-neighborhood distribution metrics in Table S1 are consistent with previously measured distributions [3-5].

S9 Figure shows the statistical distributions of the radial distance of the RyR clusters from the z-discs across all four cells. A chi-squared test showed no evidence against the hypothesis that the proportions under each histogram band are statistically similar across all four cells. Similarly, S10 Figure shows that the statistical distributions of the nearest-neighborhood distances between RyR clusters across all four cells were the same, and this result was confirmed with a chi-squared test as well.

### Model Validation

#### Re-simulating RyR cluster distributions of a cell using its observed RyR cluster distribution statistics

As a validation of our spatial statistical model of RyR cluster distributions in these cells, the radial z-disc distance distributions and the nearest-neighborhood distance function of each cell were used as input into the algorithm to re-simulate the observed distribution of the same cell. The aim of this test was to determine if the nearest-neighborhood distance and the z-disc radial distance statistics extracted from the RyR cluster distribution of a cell are sufficient to reconstruct statistically equivalent RyR cluster distributions on the myofibril geometry.

A Monte Carlo test was conducted where 99 simulations of the statistical model were generated as described in the Materials and Methods section, and we estimated F(r), G(r), K(r), and PCF(r) for each of these simulations. Using the spatial statistics extension package “spatstat” in the R statistical modeling software program, we generated envelopes for each spatial statistic, where each envelope represented the aggregate of the range of possible values that the simulated statistical model could take.

Specifically, each generated point pattern, X_s_, was placed inside an empty bounding box and 3D spatial statistics algorithms implemented in the spatstat package were used to calculate F_S_(r), G _S_(r), K _S_(r) and PCF _S_(r) as functions of the radius of the neighborhood around the reference points for which each of these measures was calculated. For each radius value, r_k_, the maximum and minimum values of each statistical measure were identified from the estimates for all of the simulations (e.g., the maximum and minimum of F_S_|r=r_k_ across all 99 simulations). Thus, maximum and minimum bounds were constructed across all r-values for which estimates of the spatial measures were calculated. These maximum and minimum bounds then formed the envelopes in which observed patterns must lie to accept that the model fits the observations (similar to S8 Figure). In choosing 99 simulations, our Monte Carlo tests have a significance level less than 1/(99+1), or α < 0.01.

S11 Figure shows the envelopes (grey shaded region) for F, G, K, and PCF for simulating the RyR cluster distribution of Cell 1 using a model based on the observed Cell 1 spatial statistics and numeric measures as detailed in the Materials and Methods. The plots show that the values of these statistics measures calculated from the observed point pattern of Cell 1 (black curve in each plot) fit well within the envelope of possible values that are taken by point patterns generated by our statistical model. Naturally, one would expect the observed G-function fits into the simulation envelope because the observed G-function is utilized as a spatial statistical measure in the energy function that must be matched by each pattern. However, F, K, and PCF can be treated as independent tests for validating our statistical model.

Testing our algorithm and statistical model on the other three cells produced similar results. These results support our hypothesis that the nearest-neighborhood distances and the radial z-disc distances of a cell’s RyR cluster point pattern are sufficient to model the spatial distribution of the RyR clusters with respect to myofibrils and z-discs in that cell.

#### Simulating RyR cluster distributions of one cell using another cell’s observed RyR cluster distribution statistics

As stated in Section 3.1, all four cells have statistically equivalent nearest-neighborhood distance and radial z-disc distance distributions. We performed additional Monte Carlo envelope tests to confirm that we could use the observed statistical distributions of the nearest-neighborhood distances and radial z-disc distances from one cell to simulate and match the observed point pattern on another cell. In essence, we tested whether there were additional characteristics that each cell exhibited that were not captured by our statistical model of RyR cluster distributions.

S12 Figure shows plots of the envelopes of G and K generated from 99 simulations of RyR cluster distributions on Cell 1 using the observed statistical distributions of Cell 2. The observed Cell 1 point pattern fits well within the possible values that can be generated by our statistical model using Cell 2 (F and PCF of the observed point pattern also fitted the corresponding envelopes, but are not shown for brevity). Simulation of RyR distribution onto the Cell 1 myofibrillar geometry based on distributions from Cell 4 statistics (not shown) further confirmed that the measured nearest-neighborhood distance and z-disc radial distance distributions are sufficient to model the RyR cluster distribution in all four cells.

#### Alternate Hypotheses

***The radial myofibril distance is not necessary for accurate simulation of RyR cluster distributions:*** The z-disc radial distance we measured is more a symptom of confocal microscopy resolution than a physical characteristic of RyR cluster locations. RyR clusters are embedded in the sarcoplasmic reticulum, a thin membrane network that envelops the myofibrils and mitochondria. This network is difficult to image with confocal techniques due to the diffraction limit and we therefore hypothesized that the RyR cluster z-disc radial distance distribution exhibits this limitation. S13 Figure shows Monte Carlo simulation-based envelopes for the RyR cluster distribution in Cell 1 that were generated by only incorporating nearest-neighborhood distance distribution and ignoring the z-disc radial distance distribution. It is clear that only the nearest-neighborhood distance and the number of clusters are necessary to model the RyR cluster distribution in cells realistically.

***The nearest-neighborhood distribution is essential to capture the variations in spatial proximity between RyR clusters:*** We hypothesized that the nearest-neighborhood distribution that we observe in the experimental data (S10 Figure) is merely a symptom of the diffraction limit as well. We removed the constraint that the nearest-neighborhood distance distributions of the observed and the simulated RyR clusters should match. Instead, we modified the simulation algorithm to enforce a minimum distance between two clusters (termed as a Gibbs hard-core model in spatial statistics theory [6]) that is equivalent to the resolvable spatial resolution of the confocal system. S14 Figure shows that this model was not sufficient to capture the spatial distribution observed since the black line is not completely contained within the statistical envelope.

### Discussion

We have presented a method to analyze and simulate the stochastic properties and anatomical spatial relationship between RyR cluster distributions and cardiac cell contractile machinery across cells excised from adult male Wistar rats.

Central to the utility of our methodology is the insight to focus on spatial co-registration with other compartments and organelles. This allows us to compare distributions (here RyR) even though cell shapes and sizes may vary greatly and is also central to the idea of computational fusion of datasets collected independently and/or using different imaging modalities, all in a format that is readily adaptable to complex model geometries.

Our study showed that the spatial statistics characteristics of an RyR cluster distribution can be accurately captured using one spatial statistical measure: nearest-neighborhood distances between RyR clusters. We determined that the nearest-neighborhood distance distribution is statistically equivalent across all four cells even though the size of the cells and the organization of the contractile machinery within them vary considerably (see S1 Figure). Realistic RyR clusters were simulated on these geometries using the measured distributions of the chosen spatial statistics measures (see S11 Figure and S12 Figure). These simulations also confirmed that the distribution of the RyR clusters was sufficiently captured by their relationship to the organization of the contractile machinery. As such, the analysis of the spatial relationship between the RyR clusters and contractile machinery helped to compare cross-cell organization.

We limited this study to examining the stochastic properties among cells from one region of a tissue block of the left ventricle of an adult male Wistar rat. Nevertheless, our measured distributions (in Table S1) were similar to those reported in previous studies on the same animal model [3,4,7].

The nearest-neighbor metric is a measure of the spread of RyR clusters. However, it could actually represent SR network branching characteristics as RyR clusters originate from this membrane network. As such, a more in-depth analysis of RyR cluster relationship to SR and myofibrils is required. Within the general philosophy of relative co-distribution of structures, it is certainly desirable to have information on as many cellular structures as possible (e.g., SR, t-tubules, mitochondria, etc.). Nevertheless, our model is able to simulate RyR clusters in relation to myofibrils (within the statistical variation found among cells in the same neighborhood) and should be sufficient to construct models of cardiac myocytes that investigate wave propagation and similar spatial aspects of cardiac calcium handling with realistic cellular geometries [8,9]. The lack of information on the SR network does not make the RyR cluster distribution model wrong. It only widens the space of possible RyR cluster distributions that can be simulated. A realistic SR network model could easily be integrated into the workflow of Fig. 8 by changing W (the window of possible RyR locations) to only contain voxels which represent a model of the SR network with respect to the contractile machinery. Thus, the incorporation of an SR network would only narrow the possible RyR cluster distributions that can be simulated.

An additional constraint was enforced on the size of the available region for RyR cluster simulation. RyR clusters could only be simulated within 0.7 μm of the myofibrillar region. This value was based on the observation that all segmented RyR clusters fell within this window. Furthermore, this constraint was only required because of the lack of accurate information on the mitochondrial region. Indeed, this restriction was removed when simulating RyR clusters on the electron tomogram because mitochondrial boundaries could be segmented and RyR clusters were restricted to the gaps between myofibrils and mitochondria.

A realistic distribution of the axial distance between RyR clusters and the z-discs was not measured in this present study due to the relatively poor quality of the z-disc stain used during data collection. We propose to collect additional confocal data where the longitudinal view of the cell is aligned with the imaging plane. This will provide high resolution measurements of the axial distance of RyR clusters to the z-discs. We would only need to extract a statistical distribution for the axial distance from such data and then use it in the presented algorithm to give more realistic distributions of the RyR clusters.

To conclude, we used this validated approach to simulate realistic RyR cluster distributions and then to generate a hybrid-spatial-scale computational model of RyR clusters, mitochondria, and myofibrils (shown in Fig. 1D and Fig. 1E, and detailed in Materials and Methods and Results in the main manuscript). This hybrid-spatial-scale model was then used to explore the effect that the structural organization of these three components of the cell have on spatio-temporal dynamics of calcium.

## References

1. Katz AM (2010) Physiology of the Heart - Arnold M. Katz. 5 ed. Lippincott Williams & Wilkins.

2. Franzini-Armstrong C (2009) Architecture and regulation of the Ca 2+delivery system in muscle cells. Appl Physiol Nutr Metab 34: 323–327. doi:10.1139/H09-017.

3. Hayashi T, Martone ME, Yu Z, Thor A, Doi M, et al. (2009) Three-dimensional electron microscopy reveals new details of membrane systems for Ca2+ signaling in the heart. J Cell Sci 122: 1005–1013. doi:10.1242/jcs.028175.

4. Soeller C, Crossman D, Gilbert R, Cannell MB (2007) Analysis of ryanodine receptor clusters in rat and human cardiac myocytes. Proc Natl Acad Sci 104: 14958–14963.

5. Jayasinghe ID, Cannell MB, Soeller C (2009) Organization of Ryanodine Receptors, Transverse Tubules, and Sodium-Calcium Exchanger in Rat Myocytes. Biophys J 97: 2664–2673. doi:10.1016/j.bpj.2009.08.036.

6. Illian J, Penttinen A, Stoyan H, Stoyan D (2008) Statistical Analysis and Modelling of Spatial Point Patterns. West Sussex: John Wiley & Sons, Ltd. 1 pp.

7. Soeller C, Jayasinghe ID, Li P, Holden AV, Cannell MB (2009) Three-dimensional high-resolution imaging of cardiac proteins to construct models of intracellular Ca2+ signalling in rat ventricular myocytes. Exp Physiol 94: 496–508. doi:10.1113/expphysiol.2008.043976.

8. Okada J, Sugiura S, Nishimura S, Hisada T (2004) Three-dimensional simulation of calcium waves and contraction in cardiomyocytes using the finite element method. AJP: Cell Physiology 288: C510–C522. doi:10.1152/ajpcell.00261.2004.

9. Wakayama Y, Miura M, Stuyvers BD, Boyden PA, Keurs Ter H (2005) Spatial nonuniformity of excitation–contraction coupling causes arrhythmogenic Ca2+ waves in rat cardiac muscle. Circ Res 96: 1266–1273.
