# Supplementary material for: Examination of the Effects of Heterogeneous Organization of RyR Clusters, Myofibrils and Mitochondria on Ca2+ Release Patterns in Cardiomyocytes
Source: PLoS Comput Biol. 2015 Sep 3;11(9):e1004417. doi: 10.1371/journal.pcbi.1004417 (PMC4559435; doi:10.1371/journal.pcbi.1004417)
Supplement: S2 Text — (DOCX) [file pcbi.1004417.s018.docx]

# S2 Text

This file includes Supplementary Text outlining the biophysical equations that were used to simulate calcium dynamics in the 3D hybrid-scale geometric model of a rat ventricular myocyte.

## Supplementary Text

### [Ca^2+^]_i_ dynamics equations

[Ca^2+^]_i_ dynamics simulations were conducted on the computational mesh (in Fig. 1) using coupled reaction-diffusion equations for free [Ca^2+^]_i_, free Fluo-4-AM [F4]_i_, and Fluo-4-bound Ca^2+^ [F4Ca]_i_:

$$\frac{\partial{[Ca}^{2+}]}{\partial t}={D_{Ca}\nabla}^{2}{[Ca}^{2+}]-J_{tnc}-J_{fluo}+J_{ryr}\text{ (1)}$$

$$\frac{\partial[F4]}{\partial t}={D_{F4}\nabla}^{2}[F4]-J_{fluo}\text{ (2)}$$

$$\frac{\partial{[F4Ca}^{2+}]}{\partial t}={D_{F4Ca}\nabla}^{2}{[F4Ca}^{2+}]+J_{fluo}\text{ (3)}$$

J_fluo_ represents the flux in the diffusing species due to calcium buffering by Fluo-4. The role of myofibrillar Troponin C as a static buffer of Ca^2+^ was incorporated as J_tnc_. The buffering rates were modeled using first-order reactions with forward and backward rates of k_i_^on^ and k_i_^off^:

$$B_{i}+ \left[ Ca \right]^{2+} \rightleftharpoons B_{i}Ca$$

J_ryr_ represents the calcium release flux from nodes describing RyR clusters. [Ca^2+^]_i_ was released from SR to cytosol at a rate of 2 pA by a prescribed release time course over a 30 ms period similar to [1-3] and defined as (see S3 Figure):

$$J_{RyR}= \left\{ \begin{aligned} 0\text{ }0<t< \boldsymbol{\tau}_{\boldsymbol{d}} ms \\ \frac{i_{Ca}\times G}{2F\times V_{cyto}} t\geq\boldsymbol{\tau}_{\boldsymbol{d}} ms \end{aligned} \right.$$

$$G= e^{\frac{t}{\tau_{1}}}(1-e^{\frac{t}{\tau_{2}}})$$

F is Faraday’s constant and V_cyto_ is the cytosolic volume into which the calcium is released. i_Ca_ represents the calcium current and G describes the exponential time course of the Ca^2+^ release, parameterized by τ_1_ and τ_2_. τ_d_ is the time point of initiation of the time course.

Each RyR cluster is triggered to release Ca^2+^ independently but with a stochastic variation in the delay as observed in calcium spark experiments [4,5]. τ_d_ is chosen for each simulated RyR cluster by randomly sampling from an exponential distribution with a characteristic decay constant of 6.7 ms as previously determined by [4].

Table S3 details the specific values for the parameters outlined above and were largely based on [3,6].

### Calcium-Induced Calcium Release Model

We tested the sensitivity of our observations of heterogeneity in the Ca^2+^ transient to Ca^2+^-dependent gating kinetics of the ryanodine receptor clusters. We developed a deterministic approximation to a two-state Markov model of RyR channels in each cluster based on [1-3,7,8].

The Ca^2+^-dependent opening and closing rate functions that represent the transitions between open and closed states of the individual RyR channels in a cluster were used to formulate an ordinary differential equation of the mean open probability of a channel during a calcium-induced calcium release event [4,5,9]:

$$\frac{dP_{open}}{dt}=kco_{ryr}\times\left( 1-P_{open} \right)-k{oc}_{ryr}\times P_{open}\left( {} \right)$$

where kco_ryr_ and koc_ryr_ are the closed-to-open and open-to-closed rate functions:

$$kco_{ryr}=30\times CF_{open}\times\frac{{Ca}_{dyad}^{4}}{K_{m}^{4}{Ca}_{dyad}^{4}}$$

$$koc_{ryr}=CF_{close}\times0.48$$

CF_close_ and CF_open_ represent the cooperativity between individual RyR channels and are dependent on the number of open channels (NumRyR_open_) and the number of closed channels (NumRyR_closed_). The total number of RyR channels is given by NumRyR and was set at 50 channels for this study. k_coop,_ set to a value of 1.0, determines the cooperativity of the channels to shut down when one of the channels transitions to the closed state.

$$CF_{open}=1+\frac{1+NumRyR_{open}}{NumRyR}$$

$$CF_{close}=k\_coop\times\left[ 1+\frac{1+NumRyR_{close}}{NumRyR} \right]$$

RyR channel sensitivity to junctional sarcoplasmic reticulum (JSR) Ca^2+^ was also accounted for in kco_ryr_ via:

$$K_{m}=6.0-0.0024\times\left[ Ca \right]_{jsr}$$

Ca^2+^ flux from JSR to dyad via the RyRs (J_ryr_), Ca^2+^ flux between network SR (NSR) and JSR, and Ca^2+^ flux from the dyad into the cytosol were defined by:

$$J_{ryr}=NumRyR\times P_{open}\times\left( Ca_{jsr}-Ca_{dyad} \right)\times g_{ryr}$$

$$J_{refill}=\left( Ca_{nsr}-Ca_{jsr} \right)\times g_{refill}$$

$$J_{cyto}=\left( Ca_{i}-Ca_{dyad} \right)\times g_{cyto}$$

where g_ryr_, g_refill_, and g_cyto_ represent rates of transfer of Ca^2+^ between the cytosolic, dyadic, and NSR and JSR compartments. Their values were based on (and set to reproduce) cluster behavior reported in prior studies [4,7,8]. Additional parameters for this model are defined in Table S3 of the main manuscript. Further details of the model can be found in [3,6-8]. We have also provided this model as a CellML file in our GitHub repository, <https://github.com/vraj004/RyR-simulator>.

## References

1. Okada J, Sugiura S, Nishimura S, Hisada T (2004) Three-dimensional simulation of calcium waves and contraction in cardiomyocytes using the finite element method. AJP: Cell Physiology 288: C510–C522. doi:10.1152/ajpcell.00261.2004.

2. Li P, Wei W, Cai X, Soeller C, Cannell MB, et al. (2009) Computational modelling of the initiation and development of spontaneous intracellular Ca2+ waves in ventricular myocytes. Philos Transact A Math Phys Eng Sci 368: 3953–3965. doi:10.1098/rsta.2010.0146.

3. Soeller C, Cannell MB (2002) Estimation of the Sarcoplasmic Reticulum Ca2+ Release FluxUnderlying Ca2+ Sparks. Biophys J 82: 2396–2414. doi:10.1016/S0006-3495(02)75584-7.

4. Wang S-Q, Song L-S, Lakatta EG, Cheng H (2001) Ca2+ signalling between single L-type Ca2+ channels and ryanodine receptors in heart cells. Nature 410: 592–596. doi:10.1038/35069083.

5. Stern MD, Ríos E, Maltsev VA (2013) Life and death of a cardiac calcium spark. The Journal of General Physiology 142: 257–274. doi:10.1085/jgp/201311034.

6. Hake J, Edwards AG, Yu Z, Kekenes-Huskey PM, Michailova AP, et al. (2012) Modelling cardiac calcium sparks in a three-dimensional reconstruction of a calcium release unit. The Journal of Physiology 590: 4403–4422. doi:10.1113/jphysiol.2012.227926.

7. Sobie EA, Dilly KW, Santos Cruz dos J, Lederer WJ, Saleet Jafri M (2002) Termination of Cardiac Ca2+ Sparks: An Investigative Mathematical Model of Calcium-Induced Calcium Release. Biophys J 83: 59–78. doi:10.1016/S0006-3495(02)75149-7.

8. Williams GSB, Chikando AC, Tuan H-TM, Sobie EA, Lederer WJ, et al. (2011) Dynamics of Calcium Sparks and Calcium Leak in the Heart. Biophys J 101: 1287–1296. doi:10.1016/j.bpj.2011.07.021.

9. Bressloff PC (2014) Stochastic Processes in Cell Biology. Springer.
