# Supplementary material for: Examination of the Effects of Heterogeneous Organization of RyR Clusters, Myofibrils and Mitochondria on Ca2+ Release Patterns in Cardiomyocytes
Source: PLoS Comput Biol. 2015 Sep 3;11(9):e1004417. doi: 10.1371/journal.pcbi.1004417 (PMC4559435; doi:10.1371/journal.pcbi.1004417)
Supplement: S1 Table — The cross-sectional area and depth are the dimensions of the field of view within the yellow rectangular windows in S1 Fig. Characteristics of the RyR cluster distribution in the four cells were calculated within this field of view. N is the number of clusters within the field of view; ρ is the density of clusters per unit volume of field of view. An explanation of z-disc radial distance can be found in S1 Text. (DOCX) [file pcbi.1004417.s019.docx]

# Table S1

RyR cluster distribution characteristics measured in the four cells in Fig. S1. The cross-sectional area and depth are the dimensions of the field of view within the yellow rectangular windows in Fig. S1. Characteristics of the RyR cluster distribution in the four cells were calculated within this field of view. N is the number of clusters within the field of view; ρ is the density of clusters per unit volume of field of view. An explanation of z-disc radial distance can be found in Text S1.

|  | **Cell 1** | **Cell 2** | **Cell 3** | **Cell 4** |
| --- | --- | --- | --- | --- |
| **Cross-sectional area (μm^2^)** | 73.8 | 103.5 | 87.3 | 120.7 |
| **Depth (μm)** | 5.2 | 5.4 | 5.4 | 5.8 |
| **Number of z-discs** | 4 | 4 | 4 | 4 |
| **N** | 328 | 518 | 520 | 686 |
| **ρ (couplons per μm^3^)** | 0.85 | 0.93 | 1.1 | 0.98 |
| **Nearest-neighbour distance mean±sd(μm)** | 0.59±0.15 | 0.57±0.15 | 0.56±0.14 | 0.57±0.14 |
| **z-disc radial distance mean±sd(μm)** | 0.26±0.14 | 0.23±0.14 | 0.25±0.14 | 0.24±0.14 |
