# Supplementary material for: Examination of the Effects of Heterogeneous Organization of RyR Clusters, Myofibrils and Mitochondria on Ca2+ Release Patterns in Cardiomyocytes
Source: PLoS Comput Biol. 2015 Sep 3;11(9):e1004417. doi: 10.1371/journal.pcbi.1004417 (PMC4559435; doi:10.1371/journal.pcbi.1004417)
Supplement: S2 Table — (DOCX) [file pcbi.1004417.s020.docx]

# Table S2

Variation in density of RyR clusters per unit cross-sectional z-disc area in each z-disc across the four cells; the values in brackets are the absolute number of RyR clusters at each z-disc.

|  | **z-disc 1** | **z-disc 2** | **z-disc 3** | **z-disc 4** |
| --- | --- | --- | --- | --- |
| **Cell 1** | 0.8 (65) | 1.1 (83) | 1.2 (88) | 1.2 (92) |
| **Cell 2** | 1.0 (99) | 1.4 (143) | 1.3 (135) | 1.4 (141) |
| **Cell 3** | 1.3 (111) | 1.6 (143) | 1.6 (136) | 1.5 (130) |
| **Cell 4** | 1.6 (190) | 1.5 (179) | 1.5 (181) | 1.1 (136) |
