# Supplementary material for: Examination of the Effects of Heterogeneous Organization of RyR Clusters, Myofibrils and Mitochondria on Ca2+ Release Patterns in Cardiomyocytes
Source: PLoS Comput Biol. 2015 Sep 3;11(9):e1004417. doi: 10.1371/journal.pcbi.1004417 (PMC4559435; doi:10.1371/journal.pcbi.1004417)
Supplement: S3 Table — Parameters were obtained from [29,38,49,50]. (DOCX) [file pcbi.1004417.s021.docx]

# Table S3

Table S3: Biochemical rate constants, diffusion constants and initial concentrations used for the Ca^2+^ simulations investigating the effects of calmodulin (CaM) and ATP buffering and Ca^2+^ dependent RyR gating. Parameters were obtained from [16,26,55,56]

| Total [CaM] | 24 μM |
| --- | --- |
| K_D_ Ca-CaM | 0.38 μM |
| [CaMCa] initial | 0.471 μM |
| [CaM] initial | 23.52 μM |
| CaM k_i_^off^ | 0.038 /ms |
| CaM k_i_^on^ | 0.1 /μMms |
| CaM Dcyto | 0.025 μm^2^/ms |
| Total [ATP] | 455 μM |
| K_D_ Ca-ATP | 0.38 μM |
| [ATPCa]_0_ | 0.318 μM |
| [ATP]_0_ | 454.682 μM |
| ATP k_i_^off^ | 45 /ms |
| ATP k_i_^on^ | 0.255 /μMms |
| ATP Dcyto | 0.14 μm^2^/ms |
| Total [CSQ] | 10000 μM |
| K_D_ Ca- CSQ | 800 μM |
| [Ca- CSQ]_0_ | 5555.5555 μM |
| [CSQ]_0_ | 4444.4445 μM |
| [Ca_NSR_]_0_ | 1000 μM |
| [Ca_JSR_]_0_ | 1000 μM |
| [Ca_dyad_]_0_ | 0.1 μM |
| g_ryr_ | 0.02 per ms |
| g_refill_ | 0.01 per ms |
| g_cyto_ | 40.0 per ms |
| NumRyR (per cluster) | 50 |
